# DCLK1 regulates stemness and IL-6/STAT3–dependent metastatic niche formation in chemoresistant ovarian cancer

**DOI:** 10.1186/s13046-026-03739-x

**Published:** 2026-05-28

**Authors:** Samrita Dogra, Sugantha Priya Elayapillai, Cole Hladik, Ameera Hasan, Maitreyee Das, Laura F. Mortan, Dongfeng Qu, Courtney W. Houchen, Bethany N. Hannafon

**Affiliations:** 1 Department of Obstetrics and Gynecology, University of Oklahoma Health Campus, 975 NE 10th St. BRC1274A, Oklahoma City, OK 73104 USA; 2 Department of Cell Biology, University of Oklahoma Health Campus, Oklahoma City, OK 73104 USA; 3 Department of Pathology, University of Oklahoma Health Campus, Oklahoma City, OK 73104 USA; 4 Department of Medicine, University of Oklahoma Health Campus, Oklahoma City, OK 73104 USA; 5 Peggy and Charles Stephenson Cancer Center, University of Oklahoma Health Campus, Oklahoma City, OK 73104 USA

**Keywords:** metastasis-initiating cells, premetastatic niche, tumor–mesothelial crosstalk, JAK–STAT signaling

## Abstract

**Background:**

Metastasis is the leading cause of cancer-related mortality. In ovarian cancer, dissemination within the peritoneal cavity is the primary driver of patient death, underscoring a critical need to define the molecular mechanisms that initiate metastatic seeding and remodel the peritoneal microenvironment. Stem-like tumor cell programs are increasingly recognized as central to metastatic competence, yet the regulators linking tumor stemness to microenvironmental reprogramming remain poorly defined. Doublecortin-like kinase 1 (DCLK1), a serine/threonine kinase associated with tumor stemness, has been implicated in cancer progression, but its role in ovarian cancer metastasis is not fully understood.

**Methods:**

We employed genetic loss-of-function approaches to investigate the role of DCLK1 in ovarian cancer metastasis using in vivo intraperitoneal dissemination models. Metastatic burden and overall survival were assessed following DCLK1 depletion. DCLK1 expression was analyzed across patient-derived ascites cultures established from primary and recurrent ovarian cancer patients and correlated with markers of metastasis-initiating cells. Functional assays were used to assess tumor cell adhesion, mesothelial clearance, stemness features and mesothelial-to-mesenchymal transition. Targeted transcriptomic and secretome analyses identified DCLK1-regulated cytokine programs. Paracrine signaling to mesothelial cells was evaluated using pharmacologic inhibition of IL-6 receptor and downstream JAK–STAT signaling.

**Results:**

Genetic loss of DCLK1 significantly reduced intraperitoneal metastatic burden and prolonged overall survival in vivo. DCLK1 expression correlated strongly with established markers of stem-like, metastasis-initiating tumor cells across the patient-derived ascites cultures. Mechanistically, DCLK1 promoted early metastatic colonization by enhancing tumor cell adhesion, mesothelial clearance, and mesothelial-to-mesenchymal transition. Transcriptomic and secretome profiling identified interleukin-6 (IL-6) as a key downstream effector of DCLK1, with DCLK1 depletion leading to a marked reduction in IL-6 expression and secretion. Tumor-derived IL-6 activated JAK–STAT signaling in mesothelial cells, promoting mesothelial migration and a permissive metastatic niche; these effects were reversed by pharmacologic blockade of IL-6 receptor signaling.

**Conclusions:**

These findings position DCLK1 as a central molecular mediator linking tumor-intrinsic stemness to microenvironmental reprogramming that primes the peritoneal niche for metastatic progression. Targeting DCLK1 or downstream IL-6/JAK–STAT signaling may therefore offer a rational strategy to disrupt metastatic dissemination in ovarian cancer.

**Supplementary Information:**

The online version contains supplementary material available at 10.1186/s13046-026-03739-x.

## Background

High-grade serous ovarian cancer (HGSOC) metastasis occurs predominantly via transcoelomic spread, a process in which exfoliated tumor cell clusters detach from the primary tumor, survive in suspension within ascites, and implant on the mesothelial lining of peritoneal organs [1, 2]. Metastatic implantation in HGSOC is increasingly recognized as a bidirectional interaction between cancer cells and the pre-metastatic niche, which is primarily composed of a mesothelial cell monolayer. Successful metastatic colonization requires tumor cells to acquire specialized survival programs, including anoikis resistance, metabolic flexibility, and the capacity to disrupt the mesothelial monolayer [3–5]. These adaptations are enriched in a rare subset of metastasis-initiating cells (MICs) that exhibit stem-like properties and seed secondary sites [6, 7]. Mesothelial cells undergo reprogramming in response to tumor-derived cytokines and extracellular vesicles, acquiring a pro-inflammatory, extracellular matrix–remodeling phenotype that facilitates mesothelial clearance and tumor adhesion [8–12].

Doublecortin-like kinase 1 (DCLK1) has emerged as a critical regulator of cancer stemness across multiple tumor types [13–15]. DCLK1 is a serine/threonine kinase and has many variants, ranging from ∼80–82 kDa long to ∼45–50 kDa short isoforms. It is best known as a marker of tuft cells and gastrointestinal cancer stem-like cells [16, 17]. DCLK1 orchestrates key pathways involved in self-renewal, epithelial–mesenchymal plasticity, and metastatic competence [18–21]. In our previous work [22], we demonstrated that DCLK1 enhances ovarian cancer cell proliferation, spheroid formation, and chemoresistance, and that elevated DCLK1 expression predicts poor patient survival. However, the role of DCLK1 in orchestrating early metastatic events, particularly tumor–mesothelial interactions and premetastatic niche formation, remains unexplored. Given the central role of secreted cytokines and chemokines in conditioning the peritoneal cavity for metastatic implantation [23], we hypothesized that DCLK1 regulates ovarian cancer metastasis by modulating tumor stemness and rewiring tumor–mesothelial crosstalk.

In this study, we demonstrate that DCLK1 is a critical mediator of metastatic progression in HGSOC. Using genetic knockout models, we show that loss of DCLK1 significantly reduces intraperitoneal metastasis and prolongs overall survival. DCLK1 expression correlates strongly with stemness and metastasis-initiating cell markers in both cell lines and patient ascites, underscoring its role in maintaining a metastatic cell state. Mechanistically, we reveal that DCLK1 promotes tumor cell adhesion to mesothelial monolayers and accelerates mesothelial clearance. We further identify a DCLK1-dependent cytokine/chemokine program, including diminished IL-6 transcription and secretion upon DCLK1 loss. Functionally, tumor-derived IL-6 activates JAK–STAT signaling in mesothelial cells, driving migration and mesothelial-to-mesenchymal transition (MMT), effects that are reversed by IL-6 receptor blockade. Together, these findings establish DCLK1 as a central regulator of stemness-driven premetastatic niche priming in ovarian cancer and highlight a targetable DCLK1–IL-6 axis that fuels metastatic progression.

## Methods

### Reagents and cell culture

Human ovarian cancer cell line OVCAR-8 CPR (cisplatin-resistant) was a generous gift from Dr. Alexander S. Brodsky (Brown University, Providence, RI). OVCAR-4 cells were a kind gift from Dr. Doris M. Benbrook (University of Oklahoma Health Campus [OUHC], Oklahoma City, OK). MeT-5A mesothelial cells were a kind gift from Dr. William L. Berry (OUHC), and LP-9 mesothelial cells were purchased from the Coriell Cell Repository. The cell lines were profiled via short tandem repeat profiling to confirm their identity. OVCAR-8 CPR and OVCAR-4 cells were cultured in RPMI media (Cytiva, SH30027.LS) supplemented with 10% fetal bovine serum (FBS; Biowest Serum Specialist, S1620-500mL) and 1% penicillin/streptomycin (Cytiva, SV30010). MeT-5A cells were grown in Medium 199 containing 1.5 g/L sodium bicarbonate (Thermo Fisher Scientific, 11150059), 10%FBS, 1% penicillin/streptomycin, 3.3 nM epidermal growth factor (EGF; Thermo Fisher Scientific, PHG0311L), 400 nM hydrocortisone (Sigma-Aldrich, H6909-10mL), 870 nM zinc-free bovine insulin (Sigma-Aldrich, I0516-5mL), and 20 mM HEPES (Cytiva, SH30237.01). LP-9 cells were cultured in a 1:1 (v/v) ratio of Ham’s F12 (Fisher Scientific, 21-127-022): Medium 199 with 1% penicillin/streptomycin, 15% FBS, 2 mmol/L L-glutamine, 10 ng/mL EGF, and 0.4 µg/mL hydrocortisone. Cells were maintained at 37 °C in a humidified atmosphere containing 5% CO2. The cell lines were tested periodically for mycoplasma using the Mycoplasma PCR Detection Kit (abm^®^, G238). If Mycoplasma was detected, mycoplasma-free stocks were used instead. Experiments were performed on cells within 15 passages post-thaw. DCLK1 inhibitor, DCLK1-IN-1 (Tocris, 7285; 10 µM); Anti-IL6 receptor antibody, Tocilizumab (Selleckchem, A2012; 1 µg/mL); Human IgG1 isotype control (Selleckchem, A2051; 1 µg/mL) were reconstituted according to the manufacturer’s protocol.

### Generation of DCLK1-modified and luciferase-tagged cell lines

OVCAR-4 cells stably overexpressing DCLK1 wild-type (WT) or kinase-domain mutants (D533N) were generated by lentiviral transduction using plenti_DCLK1 constructs (RRID: Addgene_163625, 163627) followed by blasticidin S selection. DCLK1 knockout in OVCAR-8 CPR cells was achieved using CRISPR/Cas9 sgRNAs cloned into lentiCRISPRv2 (RRID: Addgene_52961) as described previously [22]. Luciferase-tagged OVCAR-8 CPR DCLK1 knockout stable cell lines (OVCAR-8 CPR sgCtrl luc and OVCAR-8 CPR sgDCLK1 luc) were generated using lentiviral transduction of pLenti CMV V5-LUC Blast (w567-1), a gift from Eric Campeau (Addgene plasmid # 21474; RRID: Addgene_21474) [24], and blasticidin selection.

### Conditioned media collection from 3D spheroids and treatment

OVCAR-8 CPR DCLK1 knockout and OVCAR-4 DCLK1 overexpression cells (1–2 million cells) were plated on poly (2-hydroxyethyl methacrylate [HEMA])-coated plates (Sigma-Aldrich, P3932; 12 mg/mL in 95% ethanol) and cultured for 48–72 h until compact cell clusters formed. Spheroids were gently washed with prewarmed PBS and incubated in fresh low-serum (2% FBS) medium or Opti-MEM (Gibco™, 31985070) for 24–48 h. Conditioned media (CM) were collected by centrifugation at 135 × g for 5 min to remove spheroids, and the supernatant was filtered through a 0.22 μm filter. The spheroid-derived CM was used to treat MeT-5A cells for 24–48 h or stored at -80 °C until further use [25]. All CM treatments were performed under identical culture conditions to minimize variability.

### Patient ascites sample processing

Patient-derived ascites fluid was collected during paracentesis or cytoreductive surgery, in accordance with IRB-approved protocols (#15066 and #15587) and with written informed consent. Fresh ascites was transported at room temperature and processed within 1–2 h of collection. First, cellular components were separated from the ascites fluid by centrifugation at 300 × g for 5 min at room temperature. Then, red blood cells were removed from the pellet by incubation in ACK lysis buffer (Gibco™, A1049201) for 5 min at room temperature, followed by recentrifugation at 300 × g for 5 min. The cells were then plated onto poly-HEMA-coated plates and cultured in RPMI medium supplemented with matched patient acellular ascites at a 1:1 ratio. Ascites fluid collected after cell separation was centrifuged at 900 x g for 30 min, passed through a 0.8–1 μm filter, aliquoted, and stored at − 80 °C until analysis [26, 27]. Clinicopathologic characteristics of ovarian cancer patients included in this study are in Supplementary Table S1.

### Orthotopic mouse model

The animal experiments were conducted in accordance with protocols approved by the Institutional Animal Care and Use Committee at OUHC (22-044-SCHI, 25-029-SCHI). For these studies, 6-to-10-week-old female athymic nude mice (The Jackson Laboratory) were used for the following studies.

#### Endpoint study

Two million OVCAR-8 CPR sgCtrl luc and sgDCLK1#1 luc cells were plated onto 100 mm poly-HEMA-coated plates to form spheroids for 48 h. The spheroids were centrifuged to obtain a loose pellet, which was resuspended in 150 µl of sterile PBS. This spheroid suspension was injected intraperitoneally in each mouse using a 21-G needle. Mouse weight measurements and bioluminescent imaging (BLI) were performed weekly. Imaging was performed with the IVIS In Vivo Imaging System (Revvity IVIS^®^ Lumina™ LT Series III). Mice were administered D-luciferin potassium salt (100 mg/kg; Goldbio, LUCK) in sterile DPBS (Gibco, 14190-136) intraperitoneally, anesthetized using isoflurane, and then imaged. Aura Imaging Software (version 4.5.0, Spectral Instruments Imaging) was used to analyze images. Regions of interest covering the entire peritoneal cavity were selected, including tumors, and total photon counts were determined. Mice were euthanized 35 days after injection (*n* = 5 mice per group). The tumor colonies were collected and weighed. Metastatic dissemination was recorded and charted by organ system.

#### Survival study

One million OVCAR-8 CPR sgCtrl luc and sgDCLK1#1 luc cells were plated on 100 mm poly-HEMA-coated plates to form spheroids for 48 h. The spheroids were processed as described above for orthotopic implantation. Mouse weight measurements and BLI were performed weekly as described above. Survival was recorded until mice reached predefined humane endpoints based on IACUC criteria, including significant weight loss (greater than 10% of body weight), abdominal distension, impaired mobility, or signs of distress (*n* = 7 mice per group). Kaplan–Meier survival curves were generated. The maximal tumor burden was not exceeded in either of the two studies.

### Immunoblot

Western blotting was performed on whole-cell lysates (WCLs) generated from adherent, spheroids, and human patient-derived ascites cultures. WCLs were prepared using RIPA buffer (Pierce^TM^,89901) and Protease and Phosphatase Inhibitor (Pierce™, A32961). Tissue lysates were prepared from frozen tumor tissue by homogenization on ice in T-PER™ Tissue Protein Extraction Reagent (Thermo Scientific™, 78510) containing protease and phosphatase inhibitors. Protein concentration was determined using the BCA Protein Assay kit (Pierce™, 23228) and evaluated using the following methods:

#### Conventional immunoblot

Equal amounts of lysates (15–30 µg) were electrophoresed and transferred to PVDF membranes. Ponceau S Stain (Sigma-Aldrich, P7170) was used to stain total protein on the membrane to obtain a nonspecific band. Membranes were blocked in EveryBlot Blocking Buffer (Bio-Rad, 12010020) for 10 min post-transfer and incubated overnight at 4 °C with the primary antibody. After secondary antibody incubation, membranes were analyzed using Clarity Max™ Western ECL Substrate (Bio-Rad, 1705062)

#### ProteinSimple JESS

Protein expression in WCLs from patient-derived ascites cultures (~ 1 million cells) was quantified using the Jess capillary-based immunoassay system (ProteinSimple, Bio-Techne) according to the manufacturer’s protocol. Briefly, WCLs were diluted in 0.1× Sample Buffer to 1.2 mg/ml, combined with Fluorescent Master Mix at a 5:1 ratio, and heated at 95 °C for 5 min. The resulting denatured samples, total protein quantification reagents, antibody diluent, primary antibodies, HRP-linked anti-rabbit secondary antibody, and chemiluminescent detection reagents were loaded into the designated wells in the prefilled assay plates. Separation and immunodetection were performed using 12–230 kDa separation modules under default JESS settings. Compass software version 7.1 (ProteinSimple) was used for data acquisition and signal-intensity quantification. Antibody sources and dilutions are listed in Supplementary Table S2.

### Aldehyde dehydrogenase (ALDH) activity

ALDH enzymatic activity was measured using the ALDEFLUOR kit (Stemcell Technologies, 01700) according to the manufacturer’s instructions. Briefly, single-cell suspensions of OVACR-8 CPR sgCtrl and sgDCLK1 cells, and OVCAR-4 WT and OVCAR-4 DCLK1 WT cells (1–2 × 10^6^ cells/mL) were incubated with ALDEFLUOR reagent for 30–60 min at 37 °C; a matched DEAB-treated control defined the ALDH− gate [28]. The samples were analysed using a BD FACSAria™ Fusion flow cytometer (BD Biosciences).

### Proliferation assay

Proliferation assays were performed using IncuCyte^®^ S3 Live-Cell Analysis Instrument (Sartorius) and colony formation assays. For the spheroid growth assay, 1000 OVCAR-8 CPR sgCtrl luc and sgDCLK1#1 luc cells were plated in 96-well clear round bottom ultra-low attachment microplates (Corning^®^, 7007) and then placed in IncuCyte. At indicated time points, 1 image/ well for single spheroids were obtained and the brightfield area (single spheroid) was analyzed using IncuCyte 2025 A software. For the colony formation assay, 500 cells/well (OVCAR-8 CPR sgCtrl and sgDCLK1#1) were plated in 6-well plates and cultured for 10 days with media replacement every 3–4 days. On day 10, the colonies were fixed using 70% ethanol and stained with 0.4% crystal violet (CV). Images were taken using GelCount™ Colony Counter (Optronix) and quantifications were performed using GelCount Software (version 1.1.8.0) [22].

### Extreme limiting dilution assay

An extreme limiting dilution assay (ELDA) was performed to assess sphere-forming/ tumor-initiating cell frequency. OVCAR-8 CPR sgCtrl and sgDCLK1#1 single-cell suspensions were prepared and counted using trypan blue to ensure > 90% viability. Cells were plated in ultra–low-attachment 96-well plates (Corning) at serial limiting dilutions (1, 5, 10, 20, and 50 cells per well) in growth media. For each dilution, 24–48 replicate wells were seeded. Plates were incubated for 10–14 days without disturbance, and wells were scored for sphere formation (≥ 50 μm diameter) using brightfield microscopy. Sphere-forming frequency was reported as a stem cell frequency, with 95% confidence intervals, using the ELDA algorithm implemented via the online ELDA tool (http://bioinf.wehi.edu.au/software/elda/) [29].

### Cell adhesion assay

Cell adhesion assays were performed on 96-well tissue culture-treated plates coated with Fibronectin (20 µg/mL; Sigma-Aldrich, F0556), Laminin (10 µg/mL; Sigma-Aldrich, L4544), and Collagen Type I (1 mg/mL; Sigma-Aldrich, C3867). The plates were blocked with 1% BSA in PBS for 1 h at 37 °C, followed by washes with 1X PBS. Five replicates of 40,000 cells/well were plated and incubated at 37 °C for 2 h. The wells were washed thrice with 1X PBS to remove non-adherent cells, fixed with ice-cold methanol for 10 min at room temperature, and stained with 0.05% CV. The cells were destained with 10% glacial acetic acid, followed by measurement of absorbance at 570 nm, which corresponds to “adhered” cells [30].

### Quantitative real-time PCR

Total RNA was extracted from the OVCAR-8 CPR DCLK1 knockout spheroids, OVCAR-4 DCLK1 over-expression spheroids, and OVCAR-8 CPR spheroids treated with vehicle and/ or DCLK1-IN-1 (10 µM) using TRIzol reagent (Invitrogen, 15596026) and purified using Monarch RNA Cleanup Kit (New England BioLabs, T2050L). RNA concentration was quantified using the NanoDrop ND-100 Spectrophotometer (NanoDrop Technologies, Wilmington, DE, USA). qRT-PCR assays were performed using EvaGreen 2x qPCR MasterMix (Bullseye). Analysis was performed using a Bio-Rad CFX96. Primer sequences are listed in Supplementary Table S3.

### Targeted Gene Expression Profiling

Total RNA was extracted from OVCAR-8 CPR spheroids treated with DMSO (vehicle) and DCLK1-IN-1 (10 µM) for 24 h as described above. Targeted mRNA profiling was performed using the nCounter^®^ Tumor Signaling 360™ Panel (NanoString Technologies), which includes 780 genes with built-in positive, negative, and housekeeping controls covering pathways related to cellular energetics, proliferation, growth suppression, genomic instability, cell death resistance, epithelial–mesenchymal transition (EMT), and metastasis. Raw counts were processed using the ROSALIND Software version 3.16 (Rosalind) for quality control, positive-control normalization, and housekeeping gene normalization. Transcripts with counts < 20 across all samples were excluded as not expressed. Differentially expressed genes were defined as those with significant fold change (*p* < 0.05) and thresholds of < − 2 or > + 2. Group means and standard deviations (SD) were calculated for all reported genes. Additionally, differentially expressed genes identified from the NanoString analysis were further analyzed using the STRING database to assess functional interactions and pathway enrichment. Gene Ontology biological process enrichment and local network cluster enrichment were examined to identify overrepresented signaling pathways within the differentially expressed gene set [31].

### Cytokine array

The CM from OVCAR-8 CPR sgCtrl and DCLK1 knockout spheroids were examined by a human cytokine antibody array (AAH-CYT-5, RayBiotech) according to the manufacturer’s protocol. Membranes precoated with the indicated antibodies were sequentially incubated with blocking buffer, samples, detection antibodies, and washing buffers. The membranes were scanned with GenePix 4100 A Microarray Scanner (Molecular Devices). Signal intensities were quantified using GenePix^®^ Pro Microarray Acquisition and Analysis Software version 7.0 (Molecular Devices) and normalized to internal control intensities. The results are listed in Supplementary Table S4.

### Live real-time mesothelial clearance assay

Mesothelial clearance assays were performed as previously described with modifications [32, 33]. Human mesothelial cells (MeT-5A and LP-9 cells) and OVCAR-8 CPR DCLK1 knockout cells were labeled with CellTracker™ Red CMPTX (Invitrogen™, C34552) and CellTracker Green CMFDA (Invitrogen™, C7025), respectively, according to the manufacturer’s instructions. Labeled MeT-5 A and LP-9 cells (20,000–30,000 cells/ well) were seeded onto 96-well plates (MidSci, TP92096) and cultured until forming a confluent monolayer (24–48 h). Labeled OVCAR-8 CPR sgCtrl and sgDCLK1 spheroids (2000 cells/ well) were generated in 96-well ultra-low-attachment round-bottom plates (Corning, 7007) for 48 h, then gently transferred onto the mesothelial monolayer using Finntip™ Wide Orifice Pipette Tips (ThermoScientific, 9405120). After allowing spheroids to settle for 20–30 min, cultures were imaged using IncuCyte S3 Live-Imaging System (Sartorius) for 24–48 h. For drug treatment experiments, DCLK1-IN-1 (10 µM) and tocilizumab (1 µg/mL) were added either as single agents or in combination at the time of OVCAR-8 CPR spheroid transfer onto the MeT-5 A mesothelial monolayer. Phase-contrast or fluorescent images were acquired every 1–2 h to monitor tumor-induced displacement of mesothelial cells. Clearance was quantified by measuring the area of mesothelial disruption beneath each spheroid over time using ImageJ/Fiji version 1.51. The clearance index was calculated as the cleared area divided by the initial spheroid area. Only spheroids that remained intact and in contact with the monolayer for the whole imaging period were included in the analysis.

### Migration assay

The migration assays were performed using Transwell 8-µm cell culture inserts (BD Falcon, 353097). Briefly, 30,000 cells/well (MeT-5A) were plated in serum-free medium (SFM) on a transwell filter and allowed to migrate to CM derived from OVCAR-8 CPR DCLK1 knockout, OVCAR-8 CPR (± DCLK1-IN-1, 10 µM), and OVCAR-4 DCLK1 WT over-expression spheroids and patient-derived acellular ascites. After 18 h, cells from above the membrane were wiped with cotton swabs, and cells at the bottom were fixed in 10% formalin and stained with 0.05% CV. Cell migration was analyzed by counting cells using a bright-field microscope (Nikon Microscope Eclipse TE2000-U) and ImageJ version 1.51. Drugs were added to the MeT-5 A cells on top of the insert during plating.

### IL-6 Enzyme-linked immunosorbent assay (ELISA)

Human IL-6 concentration in patient-derived acellular ascites fluid (from primary and recurrent HGSOC patients) and CM from OVCAR-8 CPR DCLK1 knockout and OVCAR-4 DCLK1 over-expression spheroids were determined using a commercially available ELISA kit (RayBiotech, ELH-IL6-1). In brief, 100 µl of diluted patient-derived acellular ascites fluid or OC cell CM was added to each well in duplicate and assayed according to the manufacturer’s protocol. IL-6 concentration in the samples was determined by interpolation from the standard curve of IL-6 spanning 0–12,000 pg/ml.

### Statistical Analysis

GraphPad Prism version 10.3.0 for Windows (GraphPad Software) was used for all statistical analyses. A two-tailed unpaired Student t-test was used to compare pairs of conditions. One-way ANOVA nonparametric followed by Tukey’s/Dunnett’s post hoc test was used to compare more than two conditions. A two-way ANOVA followed by post hoc tests was used to analyze data from time-course experiments. Survival study outcomes were summarized using Kaplan–Meier curves and compared between groups using the log-rank test. Correlation analyses were performed using simple linear regression and Pearson’s correlation coefficient (r). Linear regression models were used to fit lines and compute 95% confidence intervals, and the significance of the correlation was assessed using two-tailed p-values. Correlation strength was interpreted using established thresholds (|r| ≥ 0.5, strong; 0.3–0.49, moderate; 0.1–0.3, weak). Each in vitro experiment was independent and successfully repeated at least 3 times. A P value of < 0.05 was denoted as statistical significance.

## Results

### Loss of DCLK1 attenuates metastatic progression and prolongs survival

**Fig. 1:**
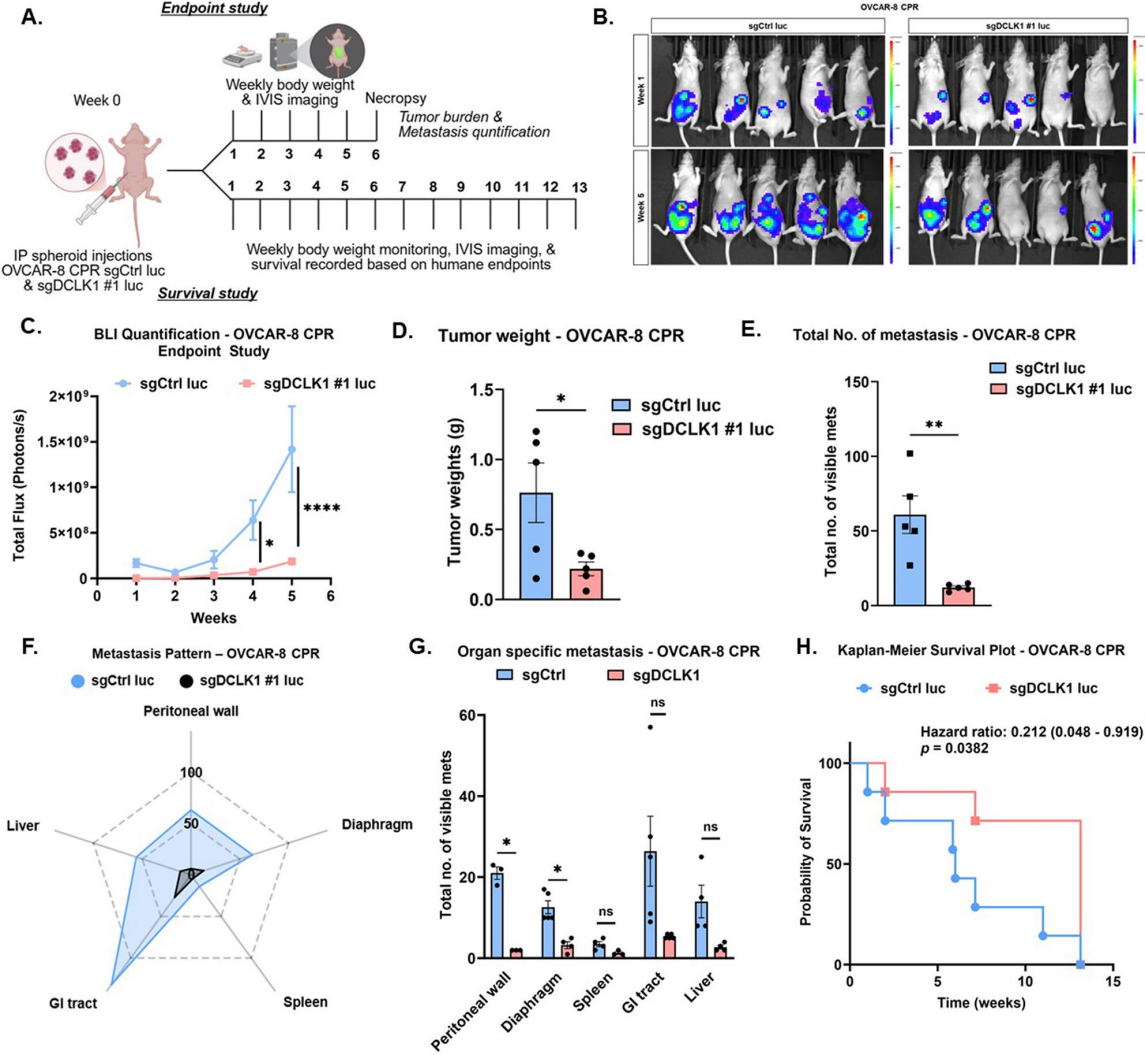
DCLK1 knockout reduced metastatic burden and improved overall survival in vivo. **A** Schematic of the endpoint and survival study performed using luc-tagged OVCAR-8 CPR DCLK1 knockout spheroids injected intraperitoneally in athymic nude mice. **B** Representative BLI of mice injected with luc-tagged OVCAR-8 CPR sgCtrl and sgDCLK1 #1 knockout spheroids, monitored longitudinally using the IVIS imaging system. **C** Quantification of total photon flux over time. **D**, **E** DCLK1 knockout reduced **D** tumor weight and **E** visible metastasis. **F** Radar plot showing metastatic distribution across different sites, wherein each spoke represents a distinct site (liver, peritoneal wall, diaphragm, GI tract, and spleen) and values represent the lesion count. **G** Total number of visible metastatic lesions in different organs. **H** Kaplan–Meier survival analysis of mice implanted with luc-tagged sgCtrl versus sgDCLK1 knockout cells. Loss of DCLK1 significantly prolonged overall survival, consistent with reduced metastatic progression. Statistical analysis was performed using a two-tailed unpaired t-test in **D**, **E**, **G**, and a log-rank (Mantel–Cox) test in H. *p < 0.05; **p < 0.01; ns: not significant. IP, Intraperitoneal; CPR, Cisplatin resistance; Luc, Luciferase; BLI, Bioluminescent imaging; GI, Gastrointestinal (includes stomach, intestines, and mesenteries)

To evaluate the functional contribution of DCLK1 to ovarian cancer dissemination, we injected tumor spheroids generated from stable luciferase-expressing OVCAR-8 CPR cells harboring CRISPR/Cas9-mediated DCLK1 knockout into the intraperitoneal cavity of mice. Disease progression was monitored longitudinally by BLI, with metastatic burden and survival quantified at experimental endpoint (Fig. 1A). Prior to in vivo injection, sgCtrl luc and sgDCLK1#1 luc spheroids did not differ significantly in average size at 48 h (Supplementary Fig. 1A-C). Passage through the syringe/needle used for intraperitoneal injection caused a decrease in spheroid size in both groups; however, this effect was not significantly different between sgCtrl luc and sgDCLK1 #1 luc spheroids (Supplementary Fig. 1B). Together, these data suggest that differences in spheroid size or injection-related disruption do not explain the DCLK1-dependent in vivo tumor phenotype. Compared with mice injected with sgCtrl spheroids, animals bearing sgDCLK1#1 spheroids exhibited a significant reduction in bioluminescent signal at weeks 4 and 5 post-implantation (Fig. 1B, C, Supplementary Table S5), indicating impaired intraperitoneal disease progression. Consistent with the imaging data, sgDCLK1 tumors displayed markedly lower tumor weights at endpoint (Fig. 1D) and a significant decrease in the total number of visible metastatic lesions in the peritoneal cavity (Fig. 1E). Metastatic nodules were predominantly detected on the peritoneal wall, diaphragm, and gastrointestinal surfaces, including the small intestine, stomach, and mesenteries, as well as the spleen and liver (Fig. 1F). Notably, sgDCLK1#1 spheroid–injected mice exhibited a pronounced reduction in metastatic nodules on the peritoneal wall and diaphragm compared with sgCtrl controls (Fig. 1F, G). Although modest decreases in metastatic burden were also observed at other organ sites such as the spleen and liver, these differences did not reach statistical significance due to high inter-animal variability (Fig. 1G). We did not observe grossly visible tumor deposits in the gonadal fat, uterine fat, or splenoportal fat within the peritoneal cavity at necropsy. Kaplan–Meier analysis revealed that mice bearing sgCtrl spheroids exhibited rapid disease progression and significantly shorter survival (media survival = 6 weeks) than mice injected with sgDCLK1#1 spheroids (median survival = 13.14 weeks), which showed improved overall survival (HR = 0.212, CI = 0.048–0.919, *p* = 0.0382; Fig. 1H). Body weights of the mice were comparable between the two groups in both studies (Supplementary Fig. 1D, E). Taken together, these data demonstrate that loss of DCLK1 diminished the miliary peritoneal deposits characteristic of advanced ovarian cancer, confirming its pro-metastatic role in driving intraperitoneal dissemination and providing a significant survival advantage.

### DCLK1 Is Associated With Metastasis-Initiating Cell Markers and Elevated ALDH1 Activity in Ovarian Cancer

**Fig. 2:**
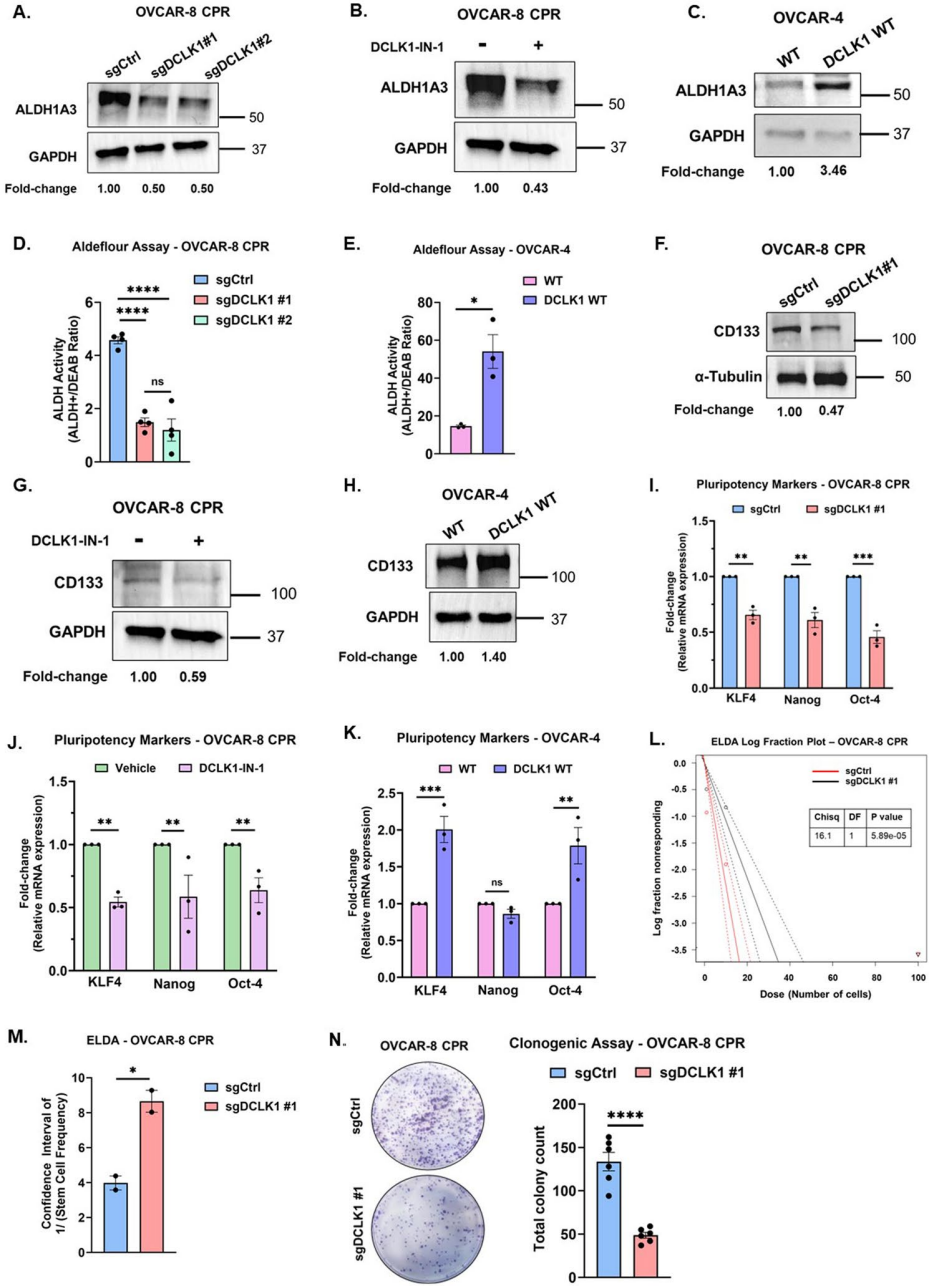
DCLK1 levels correlate with markers of metastasis-initiating cells and self-renewal capacity.** A**-**C** Immunoblot analysis showing ALDH1A3 expression in **A** OVCAR-8 CPR DCLK1 knockout spheroids, **B** OVCAR-8 CPR spheroids treated with DCLK1-IN-1, and **C** OVCAR-4 DCLK1 over-expressing spheroids. **D**,** E** Flow cytometric quantification of ALDH activity using the Aldeflour assay in **D** OVCAR-8 CPR DCLK1 knockout cells and **E** OVCAR-4 DCLK1 overexpressing cells. **F**-**H** Immunoblot analysis showing CD133 expression in **F** OVCAR-8 CPR DCLK1 knockout spheroids, **G** OVCAR-8 CPR spheroids treated with DCLK1-IN-1, and **H** OVCAR-4 DCLK1 over-expressing spheroids. **I**-**K** Expression of pluripotency-associated transcription factors (KLF4, Nanog, Oct-4) in **I** DCLK1 knockout, **J** OVCAR-8 CPR spheroids treated with DCLK1-IN-1, and **K** over-expression spheroids determined by qRT-PCR. **L**,** M** ELDA performed in low-attachment conditions to indicate **L** low tumor-initiating frequency in OVCAR-8 CPR DCLK1 knockout cells and **M** its quantification. **N** Representative images and quantification of the clonogenic assay performed in OVCAR-8 CPR DCLK1 knockout cells. These results were obtained from ≥3 independent experiments, except for ELDA (n=2 repeats), and are represented as the mean ± SEM. Statistical analysis was performed using one-way ANOVA followed by Tukey’s multiple comparison test in **D**, and two-tailed unpaired t-test in **E**,** I**,** J**,** K**,** N**. Goodness of fit tests were conducted in **L **and** M**. *p<0.05; **p<0.01; ***p<0.001; ****p<0.0001; ns: not significant. DCLK1-IN-1 (10 µM); CPR, Cisplatin resistance; ELDA, Extreme limiting dilution assay; WT, wild-type; DCLK1 WT: Over-expression of wild-type DCLK1

To determine whether DCLK1 expression correlates with stemness and metastasis-initiating cell (MIC)–associated phenotypes in ovarian cancer, we first examined its relationship with established markers of tumor-initiating potential. Genetic and pharmacological ablation of DCLK1 (using the DCLK1 inhibitor, DCLK1-IN-1 10 µM) significantly reduced aldehyde dehydrogenase (ALDH) 1A3 and 1A1 isoform levels (Fig. 2A, B; Supplementary Fig. 2A, B). Conversely, overexpression of DCLK1 wildtype (WT) resulted in a ~ 3-fold increase in ALDH1A3 levels compared with control spheroids (Fig. 2C). ALDH enzymatic activity was further assessed in the OVCAR-8 CPR DCLK1 knockout and OVCAR-4 DCLK1 over-expression systems. Basal ALDH activity differed between OVCAR-8 CPR and OVCAR-4 cells, consistent with published reports identifying OVCAR-8 as a relatively low-ALDH-activity cell line, largely associated with ALDH1A3 isoform expression, and OVCAR-4 as cell lines with higher ALDH activity [34–36]. We observed markedly decreased ALDH1 activity in DCLK1 knockout cells (Fig. 2D, Supplementary Fig. 2C), whereas DCLK1-high ovarian cancer cells exhibited significantly elevated ALDH1 activity relative to DCLK1-low cells (Fig. 2E, Supplementary Fig. 2D), consistent with an enrichment of ALDH^+^ tumor-initiating subpopulations.

We next evaluated additional MIC-associated markers. CD133 levels were reduced by ~ 0.5-fold in OVCAR-8 CPR spheroids with DCLK1 knockout and by ~ 0.6-fold following treatment with the DCLK1-IN-1 (Fig. 2F, G). Consistent with this, DCLK1 overexpression increased CD133 levels by ~ 1.4-fold relative to controls (Fig. 2H). CD44 expression was similarly diminished in both DCLK1-knockout cells and DCLK1-IN-1–treated cells (Supplementary Fig. 2E, F), indicating a strong positive correlation between DCLK1 protein levels and multiple stemness and MIC-associated markers. These observations were further supported by transcript-level associations between DCLK1 and pluripotency markers (e.g., KLF4, Nanog, and Oct-4). DCLK1 knockout led to a significant decrease in pluripotency-associated transcripts, including KLF4, Nanog, and Oct-4 (Fig. 2I). Similar findings were observed in OVCAR-8 CPR spheroids treated with DCLK1-IN-1 (10 µM) (Fig. 2J). Conversely, overexpression of DCLK1 significantly upregulated KLF4 and Oct-4 mRNA levels (Fig. 2K), demonstrating that DCLK1 expression aligns with transcriptional programs essential for tumor initiation and metastatic competence. To functionally evaluate whether DCLK1 influences self-renewal capacity, we performed extreme limiting dilution assays. The ELDA log-fraction plot demonstrated that sgDCLK1 #1 cells required more cells to form tumorspheres relative to sgCtrl cells (Fig. 2L). Further, sgDCLK1#1 cells had a higher estimated 1/stem cell frequency than sgCtrl cells, consistent with a lower frequency of tumorsphere-forming capacity after DCLK1 knockout (Fig. 2M). Furthermore, sgDCLK1#1 cells exhibited a significant reduction in colony formation relative to sgCtrl cells, collectively supporting the conclusion that DCLK1 knockout impairs self-renewal capacity (Fig. 2N). Further, western blot analysis of tumor tissues demonstrated that DCLK1 knockout tumors exhibited reduced ALDH1A3 expression relative to sgCtrl tumors, supporting a role for DCLK1 in maintaining ALDH1A3-associated tumor cell phenotypes in vivo (Supplementary Fig. S2G). Collectively, these data indicate that DCLK1 expression marks tumor cell populations enriched for stem-like and metastasis-initiating characteristics and support its functional role in maintaining tumor plasticity and self-renewal, which are required for efficient metastatic dissemination.

### Elevated DCLK1 Expression Correlates With Stemness and Metastasis-Initiating Cell Markers in Recurrent Ovarian Cancer Ascites

**Fig. 3:**
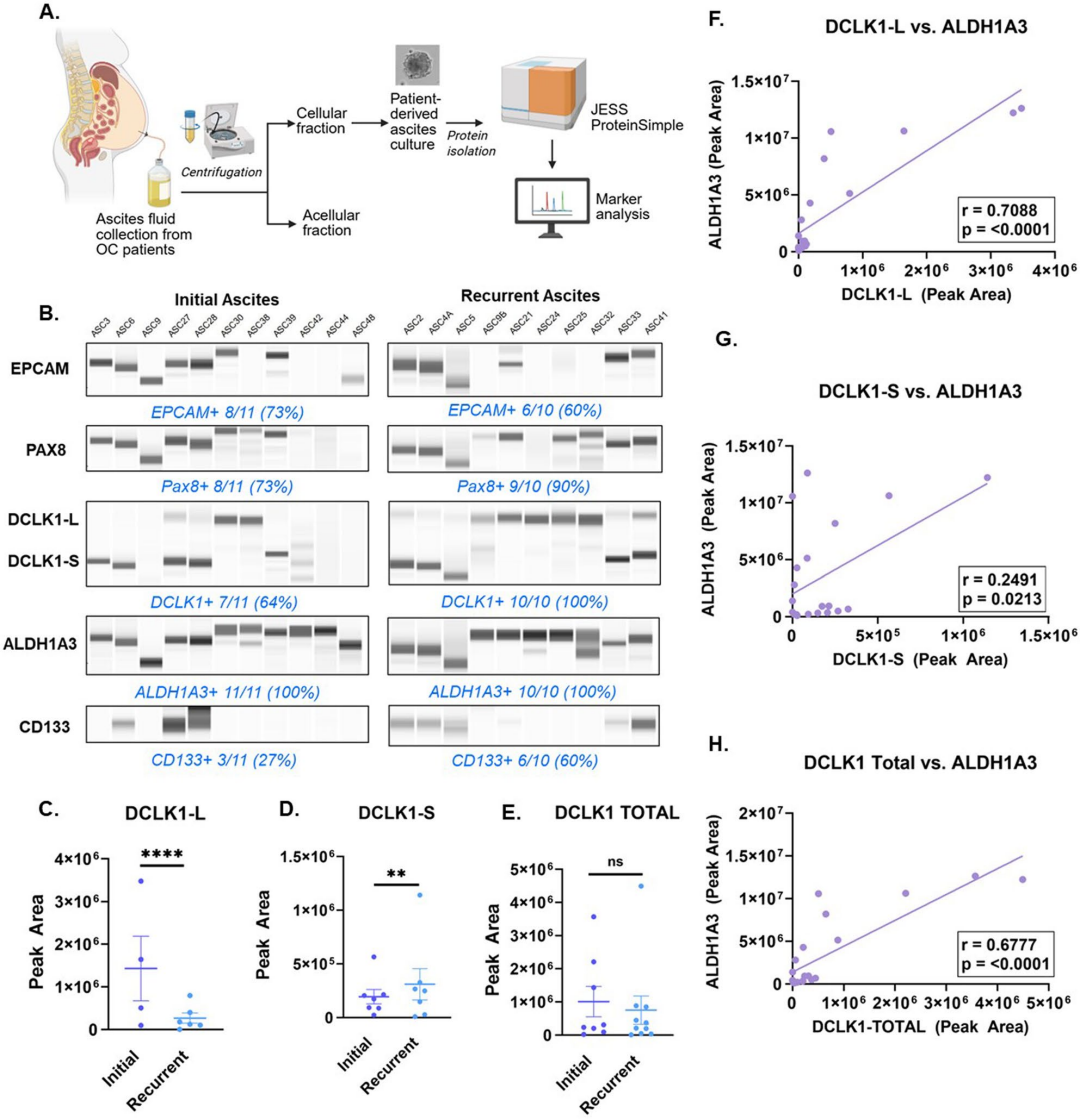
DCLK1 levels are elevated in ovarian cancer ascites and correlate with markers of metastasis, including cell markers. **A** Schematic of the workflow for ascites collection and processing for JESS ProteinSimple western blot data collection and marker analysis. **B** Representative ProteinSimple JESS electropherogram and relative frequency of occurrence of epithelial cell, DCLK1, and stem cell markers in WCL collected from PDACs established from initial (*n* = 11) and recurrent (*n* = 10) ovarian cancer ascites samples. **C**-**E** Quantification of **C** DCLK1-L, **D** DCLK1-S isoforms, and **E** total DCLK1 levels (peak area) in WCLs collected from PDACs established from initial and recurrent ovarian cancer ascites samples. **F**-**H** Correlation analysis between different isoforms, i.e., **F** DCLK1-L, **G** DCLK1-S, and **H** total DCLK1 protein levels and expression of stemness- and metastasis-initiating cell–associated marker, i.e., ALDH1A3. Statistical analysis was performed using a two-tailed unpaired t-test in C-E, and Pearson’s correlation coefficient (r) was computed in F-H. Correlation strength was interpreted as: weak (0.1–0.3), moderate (0.3–0.5), and strong (> 0.5). *p < 0.05; **p < 0.01; ***p < 0.001; ****p < 0.0001; ns: not significant; PDAC: Patient-derived ascites culture

We sought to determine whether DCLK1 expression is associated with enrichment of MIC–related signatures in patient-derived ascites. Suspended cultures were generated from ascites specimens collected from HGSOC patients at initial diagnosis and at disease recurrence. The workflow for sample processing (Fig. 3A) includes ascites centrifugation, cell isolation, and establishment of patient-derived ascites cultures (PDAC) for downstream protein characterization using JESS ProteinSimple. This platform enabled quantitative assessment of DCLK1 levels with MIC-associated markers across a panel of PDAC samples. To validate the epithelial and tumor origin of these cultures, we examined EPCAM and PAX8 expression. Approximately 70% of PDACs derived from initial ascites samples were positive for both EPCAM and PAX8. In contrast, PDACs derived from recurrent ascites exhibited broader positivity, with ~ 60% expressing EPCAM and ~ 90% expressing PAX8, reflecting the expected inter-patient heterogeneity and variable tumor cell content of ascites samples (Fig. 3B).

Next, we quantified DCLK1 expression across cells derived from initial versus recurrent ascites specimens. All recurrent PDACs (100%) expressed either the long (DCLK1-L), short (DCLK1-S), or both isoforms of DCLK1, whereas only 64% of PDACs generated from initial ascites samples were positive for DCLK1 (Fig. 3B, Supplementary Table S6). We then profiled the expression of MIC-associated markers, including ALDH1A3 and CD133. While ALDH1A3 was uniformly expressed across both initial and recurrent PDAC samples (100%), CD133 positivity increased from 27% in initial PDACs to 60% in recurrent PDACs. These findings indicate a clear enrichment of MIC-associated cell populations in recurrent, therapy-resistant PDACs (Fig. 3B).

Following peak area quantification, we observed that DCLK1-L isoform was significantly decreased in recurrent PDACs compared with those from initial ascites (Fig. 3C). In contrast, the DCLK1-S isoform was elevated considerably in the recurrent relative to the initial ascites samples (Fig. 3D). Despite these opposing isoform-specific changes, total DCLK1 levels did not differ significantly between PDACs-derived from initial and recurrent ascites samples (Fig. 3E). Correlation analyses revealed a strong positive association between DCLK1-L and total DCLK1 and ALDH1A3 a more modest but significant correlation was observed between DCLK1-S and ALDH1A3 levels (Fig. 3F-H). Together, these results suggest that the DCLK1 + PDACs are enriched for a MIC-like signature associated with enhanced metastatic potential.

### DCLK1 inactivation suppresses early peritoneal metastatic events by limiting cell adhesion and mesothelial clearance

**Fig. 4:**
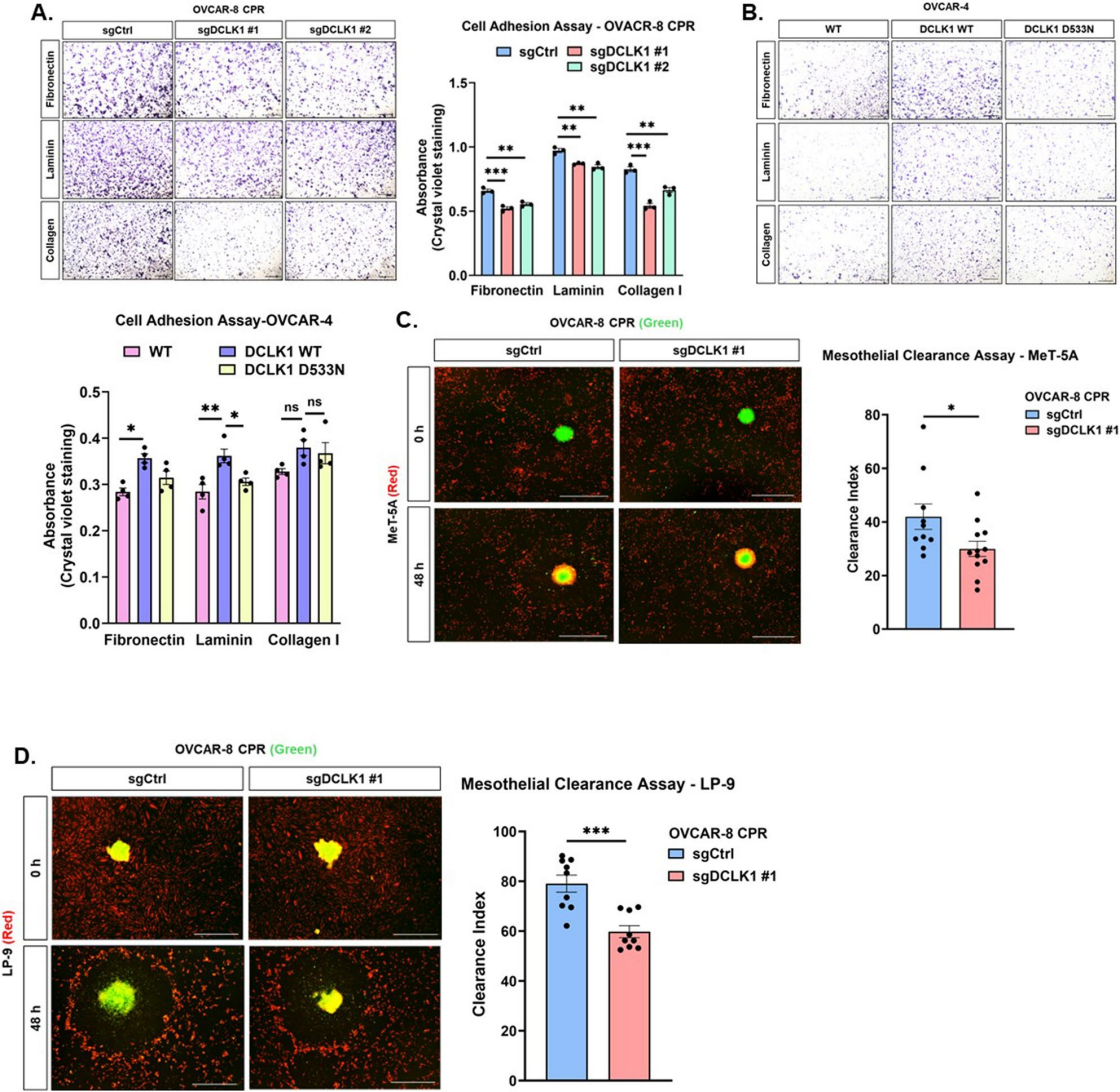
DCLK1 knockout reduces tumor cell adhesion and mesothelial cell clearance. **A** Representative images of the cell adhesion assay in OVCAR-8 CPR DCLK1 knockout cells and its quantification. **B** Representative images of cell adhesion assay in OVCAR-4 DCLK1 over-expression cells and their quantification. **C** Representative images from mesothelial clearance assays in which labeled OVCAR-8 CPR DCLK1 knockout spheroids (green) were seeded onto a labeled MeT-5 A monolayer (red), and quantification of mesothelial clearance area normalized to spheroid size. **D** Representative images from mesothelial clearance assays in which labeled OVCAR-8 CPR DCLK1 knockout spheroids (green) were seeded onto a labeled LP-9 monolayer (red) and their quantification. These results were obtained from ≥ 3 independent experiments and are represented as the Mean ± SEM. Scale bar: 500 μm for **A**, **B,** and 900 μm for **C**, **D**. Statistical analysis was performed using one-way ANOVA followed by Tukey’s multiple comparison test in **A**, **B**, and two-tailed unpaired t-test in **C**, **D**. *p < 0.05; **p < 0.01; ***p < 0.001; ns: not significant. CPR, Cisplatin resistance; WT, wild-type; DCLK1 WT: Over-expression of wild-type DCLK1; DCLK1 D533N: Over-expression of mutant DCLK1

To elucidate the mechanistic basis by which DCLK1 promotes intraperitoneal dissemination, we examined its role in early metastatic events, including tumor cell adhesion to extracellular matrix (ECM) proteins and mesothelial cell clearance. In short-term adhesion assays, OVCAR-8 CPR sgDCLK1 cells exhibited significantly reduced attachment to fibronectin, laminin, and collagen I compared to the sgCtrl cells (Fig. 4A). Conversely, DCLK1 WT over-expression in OVCAR-4 cells markedly enhanced attachment to these ECM components, although the increase in attachment to collagen I did not reach statistical significance. Notably, the over-expression of the kinase-dead DCLK1 mutant (DCLK1 D533N) abrogated this enhanced cell adhesion, indicating that DCLK1 kinase activity is required to promote tumor cell, ECM adhesion (Fig. 4B).

Successful metastatic implantation in ovarian cancer requires disruption of the mesothelial barrier. We next evaluated the clearance of a mesothelial cell monolayer using labeled mesothelial cells representative of the pleura (MeT-5A) and peritoneal wall (LP-9). DCLK1-deficient spheroids showed a pronounced impairment in their ability to clear the underlying MeT-5A cells, resulting in smaller and less-defined clearance zones relative to control spheroids (Fig. 4C). Similar results were also seen when the OVCAR-8 CPR sgDCLK1 spheroids were co-cultured with the LP-9 mesothelial cell monolayer, wherein sgDCLK1 spheroids generated a significantly smaller clearance area relative to that with sgCtrl spheroids, indicative of reduced mesothelial layer disruption (Fig. 4D). Functional assays were performed at early or controlled time points to ensure that the observed phenotypes were not confounded by differences in cell proliferation. Together, these data demonstrate that DCLK1 facilitates tumor cell adhesion and mesothelial cell clearance, two critical early steps in peritoneal metastasis that promote efficient tumor implantation.

#### DCLK1- deficiency attenuates MeT-5A migration and mesothelial-to-mesenchymal transition

**Fig. 5:**
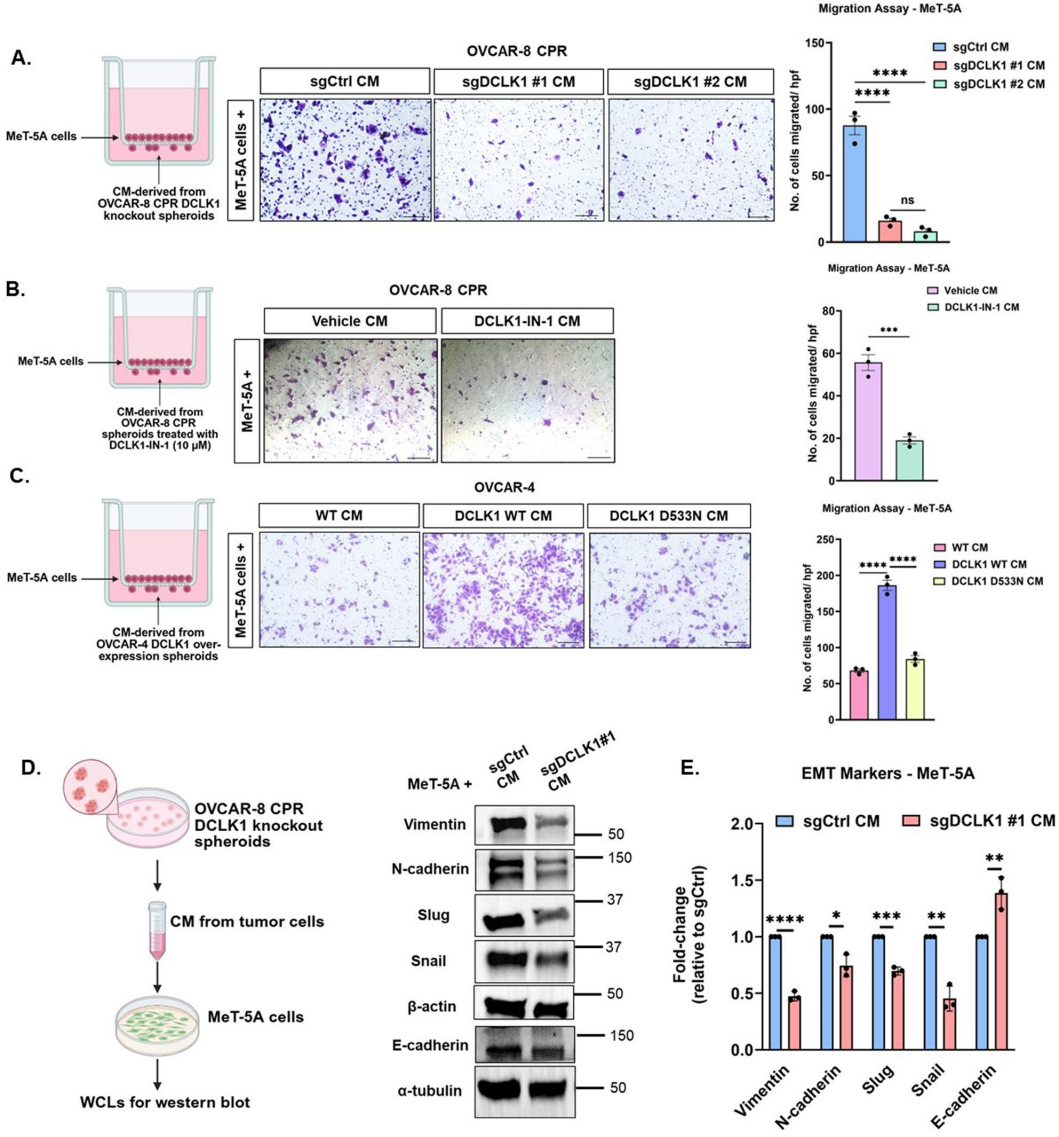
CM derived from DCLK1-expressing OC spheroids promotes MeT-5A cell migration and induces a mesothelial-to-mesenchymal transition (MMT) phenotype. **A**-**C** Schematic, representative images, and quantification of transwell migration assay using CM-derived from **A** OVCAR-8 CPR DCLK1 knock out spheroids, **B** OVCAR-8 CPR spheroids treated with DCLK1-IN-1, and **C** OVCAR-4 WT, DCLK1 WT, and DCLK1 D533N over-expression spheroids. **D**, **E** Schematic and representative western blots for EMT markers in whole cell lysates derived from MeT-5A cells grown in CM derived from OVCAR-8 CPR sgCtrl and sgDCLK1#1 spheroids, β-actin & α-tubulin - loading control **D** and its quantification **E**. These results were obtained from ≥ 3 independent experiments and are represented as the mean ± SEM. Scale bar: 500 μm for **A**-**C**. Statistical analysis was performed using one-way ANOVA followed by Sidak’s multiple comparison test in A, one-way ANOVA followed by Tukey’s multiple comparison test in **C**, and two-tailed unpaired t-test in **B**, **E**. *p < 0.05; **p < 0.01; ***p < 0.001; ****p < 0.0001; ns: not significant. DCLK1-IN-1 (10 µM). CM, Conditioned media; CPR: Cisplatin resistance; WCL, Whole-cell lysate; WT, wild-type; DCLK1 WT: Over-expression of wild-type DCLK1; DCLK1 D533N: Over-expression of mutant DCLK1

To determine the influence of tumor-intrinsic DCLK1 on mesothelial cell plasticity, we treated mesothelial cell monolayers with conditioned medium (CM) from ovarian cancer spheroids in which DCLK1 was genetically manipulated. Using transwell migration assays, we found that CM from DCLK1 knockout OVCAR-8 CPR spheroids significantly reduced mesothelial cell migration (Fig. 5A). In addition, CM from OVCAR-8 CPR spheroids treated with DCLK1-IN-1 also reduced MeT-5A migration significantly (Fig. 5B). Conversely, CM from DCLK1 WT overexpressing spheroids significantly enhanced MeT-5A cell migration (Fig. 5C). Notably, the over-expression of the kinase-dead DCLK1 mutant (DCLK1 D533N) abrogated this enhanced migratory response, indicating that DCLK1 kinase activity is required for promoting mesothelial cell migration (Fig. 5C). Note that the migration assay was performed at the 18-hour time point to ensure that the observed effects were not confounded by MeT-5A cell proliferation.

At the molecular level, exposure of MeT-5A cells to CM from DCLK1 knockout OVACR-8 CPR spheroids resulted in a significant decrease in mesenchymal markers, including Vimentin, N-cadherin, Slug, and Snail, accompanied by a concomitant increase in the epithelial marker, E-cadherin (Fig. 5D, E). These changes indicate suppression of mesothelial-to-mesenchymal transition (MMT) upon DCLK1 inactivation in tumors. Together, these data suggest that tumor-intrinsic DCLK1 modulates the mesothelial cell behavior in a paracrine manner by inducing MMT and enhancing mesothelial cell migration, thereby creating a more permissive niche for ovarian cancer metastasis within the peritoneal cavity.

### Tumor-intrinsic DCLK1 regulates a pro-metastatic cytokine- and chemokine-secretory program

**Fig. 6:**
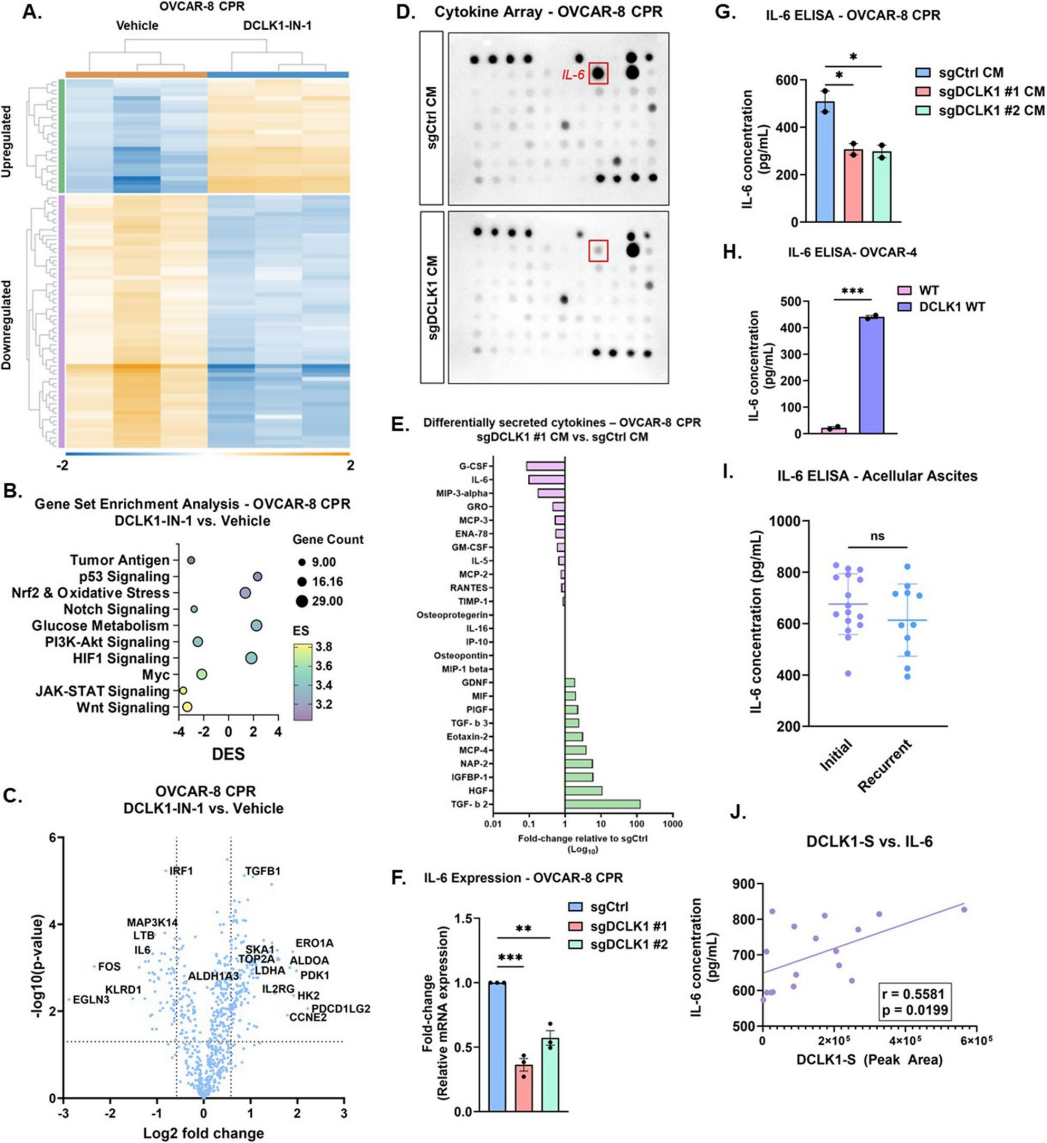
DCLK1 expression modulates IL-6 levels in conditioned media derived from tumor cells. **A** Heat map of the normalized data, scaled to give all genes equal variance, was generated via unsupervised clustering in OVCAR-8 CPR spheroids treated with DCLK1-IN-1 (10 µM) for 24 h. **B** Bubble plot summarizing statistically significant GSEA results, where each bubble represents curated pathways, bubble size represents gene set size, and bubble color denotes statistical significance. **C** Volcano plot displaying each gene’s −log10 (p-value) and log2 fold change with the selected covariate. Statistically significant genes fall above the horizontal line, and highly differentially expressed genes fall to either side. The horizontal line indicates p-value < 0.05. **D** Representative cytokine array membranes showing differential secretion of cytokines and chemokines in CM derived from OVCAR-8 CPR sgCtrl and sgDCLK1 #1 spheroids. **E** Quantification of spot intensities, normalized to internal controls, and fold changes calculated relative to sgCtrl CM. **F** Expression of IL-6 mRNA in OVCAR-8 CPR DCLK1 knockout spheroids as quantified by qRT-PCR. G-I. Quantification of secreted IL-6 in CM-derived from **G** OVCAR-8 CPR DCLK1 knockout spheroids, **H** OVCAR-4 DCLK1 over-expressing spheroids, and **I** acellular ascites derived from initial (*n* = 17) and recurrent (*n* = 11) ascites samples from OC patients. **J** Correlation analysis between DCLK1-S isoform and IL-6 concentration. Statistical analysis was performed using one-way ANOVA followed by Tukey’s multiple comparison test in **F**, **G**; two-tailed unpaired t-test in **H**, **I**, and Pearson’s correlation coefficient (r) was computed in **J**. Correlation strength was interpreted as: weak (0.1–0.3), moderate (0.3–0.5), and strong (> 0.5). *p < 0.05; **p < 0.01; ***p < 0.001; ****p < 0.0001; ns: not significant. CPR, cisplatin resistant; CM, conditioned-media; WT, wild-type; DCLK1 WT: Over-expression of wild-type DCLK1

To gain mechanistic insight into how DCLK1 modulates mesothelial cell behavior to promote metastasis, we next examined alterations in the tumor signaling pathways and secretory programs downstream of DCLK1. Transcriptomic profiling of 780 tumor signaling genes spanning more than 40 cancer-associated pathways was performed in OVCAR-8 CPR spheroids treated with the DCLK1 inhibitor DCLK1-IN-1 (10 µM for 24 h). Unsupervised hierarchical clustering of normalized expression data demonstrated strong reproducibility across biological replicates (Fig. 6A). Pharmacologic inhibition of DCLK1 attenuated signaling pathways implicated in tumor progression and metastasis, including Wnt, JAK-STAT, Myc, stemness, and PI3K-AKT pathways. In contrast, pathways associated with NRF2 signaling, oxidative stress responses, and glucose metabolism were upregulated (Fig. 6B, C). Consistent with the gene set enrichment analysis, STRING Gene Ontology biological process enrichment showed enrichment of cytokine-mediated signaling pathways. In addition, STRING local network cluster enrichment identified the JAK–STAT signaling pathway, further supporting a role for inflammatory cytokine signaling within the DCLK1-regulated transcriptional program (Supplementary Fig. 3A, B). To determine whether DCLK1 regulates the tumor secretome, we performed cytokine array analysis using CM derived from OVCAR-8 CPR sgCtrl and sgDCLK1 spheroids. Loss of DCLK1 reduced the secretion of several pro-tumorigenic cytokines and chemokines, including IL-6, G-CSF, MIP-3α, and GRO, whereas an increase in the secretion of TGF-β2, HGF, and IGFBP-1 was observed (Fig. 6D, E).

Given the established role of IL-6 in promoting metastasis, we further examined the relationship between DCLK1 and IL-6 signaling. IL-6 mRNA levels were significantly reduced in DCLK1 knockout spheroids compared with control (Fig. 6F). Consistent with this transcriptional regulation, ELISA-based quantification revealed that OVCAR-8 CPR sgDCLK1 spheroids secreted significantly less IL-6 into the CM relative to sgCtrl spheroids (Fig. 6G, Supplementary Fig. 3C), whereas DCLK1 WT over-expression significantly enhanced IL-6 secretion (Fig. 6H). We next quantified IL-6 levels in acellular ascites fluid obtained from patients with initial and recurrent ovarian cancer. IL-6 was detectable in all ascites samples, with concentrations ranging from approximately 350–850 pg/mL, reflecting substantial inter-patient heterogeneity (Supplementary Fig. 3D, E). Although no statistically significant difference in IL-6 concentration was observed between initial and recurrent ascites samples (Fig. 6I), correlation analysis revealed a significant positive association between DCLK1-S isoform and IL-6 concentration (Fig. 6J). On the contrary, DCLK1-L levels were negatively correlated with IL-6 concentration, while total DCLK1 exhibited only a weak positive correlation (Supplementary Fig. 3F, G). Collectively, these data indicate that DCLK1, particularly the short isoform, is associated with IL-6 production and secretion, supporting a paracrine mechanism whereby DCLK1-S-high tumor cells influence mesothelial cell behavior to promote metastatic niche formation.

### DCLK1-driven paracrine IL-6 signaling engages mesothelial JAK–STAT3 to promote metastasis

**Fig. 7:**
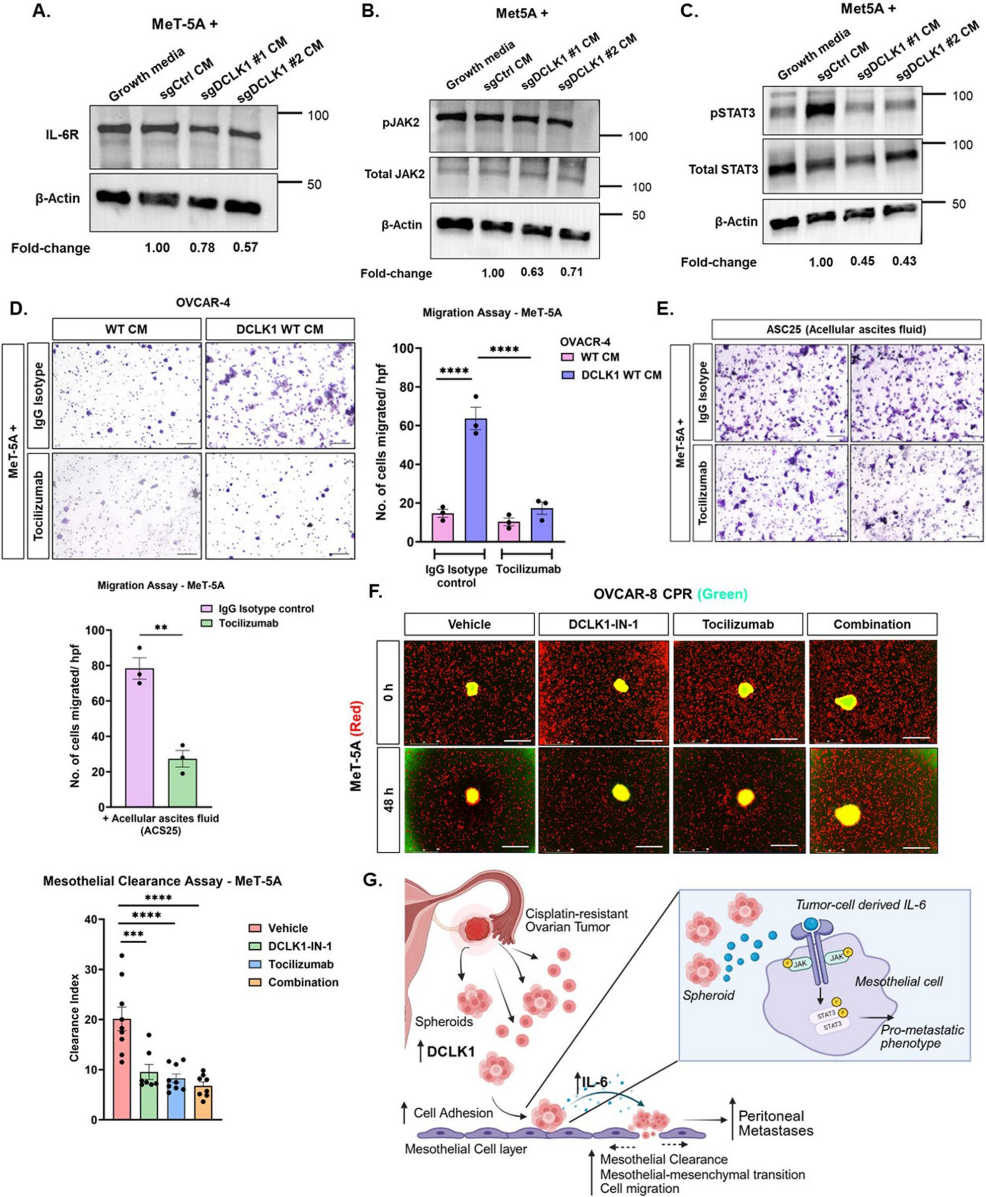
Paracrine IL-6 signaling activates mesothelial JAK-STAT3 pathway to facilitate DCLK1-dependent metastasis. **A** Representative western blot for IL-6 receptor expression in WCLs derived from MeT-5 A cells treated with CM from OVCAR-8 CPR DCLK1 knockout spheroids. **B** Immunoblot analysis of phosphorylated JAK2 in MeT-5 A cells following CM treatment from OVCAR-8 CPR sgCtrl and sgDCLK1 spheroids. Total JAK2 and β-Actin served as loading controls. **C** Representative western blot for phospho STAT3 in WCLs derived from MeT-5 A cells treated with CM from OVCAR-8 CPR DCLK1 knockout spheroids. Total STAT3 and β-Actin served as loading controls. **D** Representative images and quantification of MeT-5 A cell migration following treatment with CM derived from OVCAR-4 DCLK1–overexpressing spheroids, with or without tocilizumab (IL-6R–blocking antibody). **E** Representative images and quantification of MeT-5 A cell migration following treatment with acellular ascites fluid derived from a recurrent OC patient positive for DCLK1 expression and IL-6 levels, with or without tocilizumab (IL-6R–blocking antibody). **F** Representative images and quantification of mesothelial clearance assay in which labeled OVCAR-8 CPR spheroids (green) were seeded onto a labeled MeT-5 A monolayer (red) in presence of DCLK1-IN-1 and tocilizumab as single agents and in combination. **G** Schematic representation of the proposed model in which DCLK1-high OC spheroids secrete IL-6, reshaping the peritoneal microenvironment to support metastatic spread. Tumor-derived IL-6 activates JAK–STAT3 signaling in adjacent mesothelial cells, inducing mesothelial-to-mesenchymal transition, enhanced migration, cell adhesion, and mesothelial clearance. These results were obtained from ≥ 3 independent experiments and are represented as the Mean ± SEM. Statistical analysis was performed using one-way ANOVA followed by Tukey’s multiple comparison test in **D**, **F**, and two-tailed unpaired t-test in E. **p < 0.01; ****p < 0.0001. CM, conditioned media; WT, wild-type; DCLK1 WT: Over-expression of wild-type DCLK1

Given the observed DCLK1-dependent effect on IL-6 secretion, we next investigated whether tumor-derived IL-6 activates JAK–STAT3 signaling in mesothelial cells through a paracrine mechanism. Exposure of MeT-5 A cells to CM-derived from DCLK1 knockout spheroids resulted in a 0.5-0.8-fold reduction in IL-6 receptor (IL-6R) expression compared with CM from control spheroids (Fig. 7A, Supplementary Fig. 4A). This was accompanied by a 0.6-0.7-fold decrease in JAK2 phosphorylation and a subsequent ~ 0.4-fold reduction in STAT3 activation, indicating that tumor-intrinsic DCLK1 is required to engage the mesothelial JAK2-STAT3 signaling (Fig. 7B, C, Supplementary Fig. 4B, C). Significantly, pharmacologic blockade of IL-6R-signaling using the neutralizing antibody Tocilizumab significantly reduced MeT-5 A cell migration in the presence of CM from DCLK1 WT-overexpressing OVCAR-4 spheroids relative to CM from control spheroids (Fig. 7D, Supplementary Fig. 4D). Similarly, Tocilizumab treatment markedly impaired MeT-5 A migration in response to acellular ascites from a recurrent ovarian cancer patient (ASC25) positive for DCLK1 and IL-6 (Fig. 7E, Supplementary Table S6), confirming that IL-6 is a key mediator of this paracrine signaling axis. Further, combining DCLK1-IN-1 with Tocilizumab was effective in reducing mesothelial cell clearance relative to vehicle control (Fig. 7F). Collectively, these findings demonstrate that tumor-intrinsic DCLK1 modulates IL-6 secretion, which, in turn, activates the JAK–STAT3 signaling axis in mesothelial cells in a paracrine manner, remodeling the mesothelial compartment to facilitate early metastatic niche formation and peritoneal dissemination (Fig. 7G).

## Discussion

High-grade serous ovarian cancer (HGSOC) is characterized by rapid peritoneal dissemination and frequent development of therapy-resistant recurrent disease. Identifying molecular drivers of metastatic colonization and the premetastatic niche is therefore critical to improving patient outcomes. In this study, we demonstrate that the stem cell–associated kinase DCLK1 functions as a central regulator of metastatic progression in ovarian cancer through coordinated effects on tumor-intrinsic stemness, early metastatic events, and paracrine modulation of the mesothelial microenvironment. DCLK1 knockout reduced peritoneal metastasis and improved overall survival in vivo following spheroid injection, suggesting that this phenotype is not explained by gross differences in spheroid size alone. However, DCLK1 loss may alter other spheroid properties, such as cohesivity and structural organization, that influence tumor implantation and mesothelial clearance. Importantly, the improved overall survival observed in mice injected with DCLK1-knockout spheroids paralleled our previous clinical findings showing that low DCLK1 expression is associated with increased survival in ovarian cancer patients following platinum-based therapy [22]. Together, these findings support a broader role for DCLK1 in regulating multicellular behaviors that drive metastatic progression in the peritoneal cavity. We show that DCLK1 expression correlates with metastasis-initiating cell (MIC) markers, including ALDH1A3 and CD133, in both tumor spheroids and patient-derived ascites organoids. Notably, the short isoform of DCLK1 (DCLK1-S) was associated with elevated MIC-like signatures. Functional assays confirmed that DCLK1 promotes tumor cell self-renewal and tumorsphere formation, supporting its role in sustaining stem-like populations with high metastatic potential. These findings align with previous reports implicating DCLK1 in cancer stemness across multiple tumor types [37–39]. In addition, DCLK1-S high expression has been associated with increased invasive potential in human colorectal cancer [40], which supports our findings in the context of ovarian cancer metastasis. The distinct roles of DCLK1 isoforms, together with the enrichment of DCLK1-S in recurrent ascites, support the possibility of isoform-specific contributions to MIC enrichment and paracrine signaling, potentially arising through alternative promoter switching. Prior studies in colon cancer showed that β-catenin/TCF4/LEF activates the α-promoter, whereas NF-κB p65 activates the β-promoter to drive DCLK1-S expression [41]. In parallel, recent work in clear-cell renal cell carcinoma identified hypoxia–HIF2α–PLOD2 signaling as an activator of the α-promoter, leading to increased DCLK1-L expression [38]. These observations suggest that similar regulatory mechanisms may underlie isoform-specific DCLK1 expression and function in ovarian cancer, an area warranting further mechanistic investigation. Fibronectin, laminin, and collagen I are crucial extracellular matrix (ECM) proteins that actively promote ovarian cancer metastasis. Collagen I forms dense matrices that ovarian cancer cells prefer. At the same time, fibronectin facilitates cell spreading and movement, and laminin promotes invasive phenotypes, all of which interact with integrin receptors to drive the metastatic cascade from the primary tumor to distant sites such as the peritoneum [42–44]. We demonstrate that DCLK1 enhances early metastatic events, including tumor cell adhesion to ECM components and mesothelial cell clearance. Loss of DCLK1 impaired adhesion to fibronectin, laminin, and collagen I and significantly reduced spheroids’ ability to disrupt mesothelial monolayers. These observations underscore the critical role of DCLK1 in facilitating peritoneal implantation, a rate-limiting step in ovarian cancer dissemination [45, 46]. Importantly, DCLK1 kinase activity was required to promote adhesion, suggesting that its enzymatic activity could be a therapeutic target.

Beyond tumor-intrinsic effects, our data reveal that DCLK1 modulates the tumor secretome, thereby influencing mesothelial cell behavior. Conditioned media from DCLK1 knockout spheroids suppressed MMT and reduced mesothelial migration, whereas high DCLK1 promoted mesothelial migration. Cytokine array and targeted transcriptomic analyses identified IL-6 as a key mediator of this paracrine signaling. DCLK1 knockout decreased IL-6 expression and secretion, whereas DCLK1 overexpression increased IL-6 production. Functional blockade of IL-6 signaling via a neutralizing IL-6 receptor antibody (Tocilizumab) attenuated JAK–STAT3 activation in mesothelial cells and reduced mesothelial migration, thereby establishing a DCLK1–IL-6–JAK–STAT3 axis that remodels the mesothelial compartment to create a permissive niche for metastatic implantation.

This study has several limitations, including the relatively small patient cohort and lack of longitudinal analysis. Future work will validate these findings in larger, more diverse patient cohorts and incorporate serial patient sampling to monitor DCLK1 expression and IL-6 levels over time within the same individuals, thereby providing a more dynamic view of their evolution during disease progression and recurrence. In addition, although our study uses the widely adopted OVCAR-8 and OVCAR-4 ovarian cancer models, validation in additional cell lines will be an important direction for future studies. Nevertheless, our findings integrate tumor-intrinsic stemness with microenvironmental modulation to provide a mechanistic framework for DCLK1-dependent ovarian cancer metastasis. Prior work from our group demonstrated that DCLK1 knockout restores cisplatin sensitivity and that combining a DCLK1 inhibitor with cisplatin reduces tumor growth, supporting the therapeutic relevance of DCLK1 targeting in chemoresistant disease. Building on this, the current study identifies IL-6/JAK–STAT3 signaling as an additional associated vulnerability. Our findings provide a strong rationale for future preclinical studies evaluating combined inhibition of DCLK1 kinase activity and IL-6–driven mesothelial remodeling, which may represent a dual therapeutic strategy to limit both ovarian cancer cell dissemination and the establishment of metastatic niches. Several studies have shown that IL-6/STAT3 signaling promotes chemoresistance and EMT in ovarian cancer [47–49]. In addition, activation of the JAK2 and STAT3 pathway has also been associated with cancer stem cell-like phenotype (increased CD44 and Nanog levels) in ovarian cancer, which can be targeted using CYT387 (JAK2-specific small molecule inhibitor), indicating that the JAK2-STAT3 pathway is one of the drivers of stemness in ovarian cancer [50, 51]. Several clinical trials have examined the use of monoclonal antibodies targeting the IL-6 pathway in the treatment of ovarian cancer. Overall, these studies have shown limited promise for the utility of IL-6 pathway inhibition as a standalone treatment for ovarian cancer, while supporting the possibility that novel combination regimens could improve patient outcomes [52].

## Conclusions

In conclusion, our study establishes DCLK1 as a critical driver of ovarian cancer metastasis through dual mechanisms: promoting tumor cell stemness and facilitating early metastatic events, including adhesion and mesothelial cell clearance. Additionally, DCLK1 orchestrates a pro-metastatic tumor secretome, with IL-6 acting in a paracrine manner to activate JAK–STAT3 signaling in mesothelial cells, thereby remodeling the peritoneal niche to support metastatic implantation. Isoform-specific contributions, particularly those of DCLK1-S, in recurrent ascites further highlight its role in therapy-resistant disease. Collectively, these findings identify DCLK1 as a promising therapeutic target to disrupt both tumor-intrinsic and microenvironment-mediated mechanisms of ovarian cancer dissemination.

## Supplementary Information

Supplementary Material 1. Supplementary Table S1. Clinicopathologic Characteristics of Ovarian Cancer Patients in this Study.Supplementary Table S2. Antibody dilutions used for Western blots.Supplementary Table S3. Primer sequences for qRT-PCRSupplementary Table S4. Differentially expressed cytokines/ chemokines.Supplementary Table S5. DCLK1 Expression in ovarian cancer patient-derived ascites cultures

Supplementary Material 2. Supplementary Figure 1. In vivo DCLK1 knockout has no significant effect on body weight in OC mouse modelsSupplementary Figure 2. Loss of DCLK1 reduces ALDH1A1/CD44 expression and ALDH activity.Supplementary Figure 3. DCLK1 expression is correlated with IL-6 secretion in patient-derived acellular ascites samples.Supplementary Figure 4. Paracrine IL-6 signaling activates mesothelial JAK–STAT3 signaling.

## Data Availability

The sequencing data generated in this study are publicly available in the Gene Expression Omnibus (GEO) at GSE333343.” Other data will be made available upon a reasonable request.

## Abbreviations

ALDH: Aldehyde dehydrogenase
BLI: Bioluminescent imaging
DCLK1: Doublecortin—like kinase 1
ECM: Extracellular matrix
EGF: Epidermal growth factor
ELDA: Extreme limiting dilution assay
EMT: Epithelial—mesenchymal transition
EPCAM: Epithelial cell adhesion molecule
G: CSF—granulocyte colony—stimulating factor
GRO: Growth—regulated oncogene (chemokine family)
HGF: Hepatocyte growth factor
HGSOC: High—grade serous ovarian cancer
HR: Hazard ratio
IL-6: interleukin 6
IL-6R: interleukin—6 receptor
JAK2: Janus kinase2
JAK-STAT: Janus kinase—signal transducer and activator of transcription
MICs: Metastasis—initiating cells
MIP: 3α—macrophage inflammatory protein 3 alpha (CCL20)
MMT: Mesothelial—to—mesenchymal transition
NRF2: Nuclear factor erythroid 2—related factor 2
PDAC(s): Patient—derived ascites culture(s)
STAT3: Signal transducer and activator of transcription 3
TGF: β2—transforming growth factor beta 2

## References

[1] Deng M, Yang R, Jiang J, Zhang J, He J, Miao J. The silent spread: exploring diverse metastatic pathways in high-grade serous ovarian cancer. Front Med (Lausanne). 2025;12:1539024. 40109727 10.3389/fmed.2025.1539024 PMC11919666

[2] Ritch SJ, Telleria CM. The Transcoelomic Ecosystem and Epithelial Ovarian Cancer Dissemination. Front Endocrinol (Lausanne). 2022;13:886533. 35574025 10.3389/fendo.2022.886533 PMC9096207

[3] Qian J, LeSavage BL, Hubka KM, Ma C, Natarajan S, Eggold JT et al. Cancer-associated mesothelial cells promote ovarian cancer chemoresistance through paracrine osteopontin signaling. J Clin Invest. 2021;131(16):e146186. 10.1172/JCI146186 PMC8363279 34396988

[4] Drapela S, Gomes AP. Metabolic requirements of the metastatic cascade. Curr Opin Syst Biol. 2021;28:100381. 10.1016/j.coisb.2021.100381 PMC8535854 34693082

[5] Simpson CD, Anyiwe K, Schimmer AD. Anoikis resistance and tumor metastasis. Cancer Lett. 2008;272(2):177–85. 18579285 10.1016/j.canlet.2008.05.029

[6] Celia-Terrassa T, Kang Y. Distinctive properties of metastasis-initiating cells. Genes Dev. 2016;30(8):892–908. 27083997 10.1101/gad.277681.116 PMC4840296

[7] Wang J, Ford JC, Mitra AK. Defining the Role of Metastasis-Initiating Cells in Promoting Carcinogenesis in Ovarian Cancer. Biology (Basel). 2023;12(12):1492. 10.3390/biology12121492 PMC10740540 38132318

[8] Mutsaers SE, Prele CM, Pengelly S, Herrick SE. Mesothelial cells and peritoneal homeostasis. Fertil Steril. 2016;106(5):1018–24. 27692285 10.1016/j.fertnstert.2016.09.005

[9] Matte I, Lane D, Bachvarov D, Rancourt C, Piche A. Role of malignant ascites on human mesothelial cells and their gene expression profiles. BMC Cancer. 2014;14:288. 24761768 10.1186/1471-2407-14-288 PMC4008408

[10] Bilandzic M, Stenvers KL. Assessment of ovarian cancer spheroid attachment and invasion of mesothelial cells in real time. J Vis Exp. 2014(87):51655. 10.3791/51655 PMC4199467 24893837

[11] Shishido A, Mori S, Yokoyama Y, Hamada Y, Minami K, Qian Y, et al. Mesothelial cells facilitate cancer stem–like properties in spheroids of ovarian cancer cells. Oncol Rep. 2018;40(4):2105–14. 30066911 10.3892/or.2018.6605

[12] Lopez-Cabrera M. Mesenchymal Conversion of Mesothelial Cells Is a Key Event in the Pathophysiology of the Peritoneum during Peritoneal Dialysis. Adv Med. 2014;2014:473134. 26556413 10.1155/2014/473134 PMC4590954

[13] Chhetri D, Vengadassalapathy S, Venkadassalapathy S, Balachandran V, Umapathy VR, Veeraraghavan VP, et al. Pleiotropic effects of DCLK1 in cancer and cancer stem cells. Front Mol Biosci. 2022;9:965730. 36250024 10.3389/fmolb.2022.965730 PMC9560780

[14] Chandrakesan P, Panneerselvam J, May R, Weygant N, Qu D, Berry WR, et al. DCLK1-Isoform2 Alternative Splice Variant Promotes Pancreatic Tumor Immunosuppressive M2-Macrophage Polarization. Mol Cancer Ther. 2020;19(7):1539–49. 32371580 10.1158/1535-7163.MCT-19-0776 PMC7883901

[15] Chandrakesan P, Weygant N, May R, Qu D, Chinthalapally HR, Sureban SM, et al. DCLK1 facilitates intestinal tumor growth via enhancing pluripotency and epithelial mesenchymal transition. Oncotarget. 2014;5(19):9269–80. 25211188 10.18632/oncotarget.2393 PMC4253433

[16] Cao Z, Weygant N, Chandrakesan P, Houchen CW, Peng J, Qu D. Tuft and Cancer Stem Cell Marker DCLK1: A New Target to Enhance Anti-Tumor Immunity in the Tumor Microenvironment. Cancers (Basel). 2020;12(12):3801. 10.3390/cancers12123801 PMC7766931 33348546

[17] Nakagawa A, Von Alt KN, Mino-Kenudson M, Adams CE, Purschke B, Taniue K et al. De novo formation of tuft and goblet cells protects the intrapancreatic biliary duct system from inflammatory injury. FEBS J. 2026;293(7):2133–54. 10.1111/febs.70330 41241846

[18] Vega KJ, May R, Sureban SM, Lightfoot SA, Qu D, Reed A, et al. Identification of the putative intestinal stem cell marker doublecortin and CaM kinase-like-1 in Barrett’s esophagus and esophageal adenocarcinoma. J Gastroenterol Hepatol. 2012;27(4):773–80. 21916995 10.1111/j.1440-1746.2011.06928.x PMC3289765

[19] Kalantari E, Ghods R, Saeednejad Zanjani L, Rahimi M, Eini L, Razmi M, et al. Cytoplasmic expression of DCLK1-S, a novel DCLK1 isoform, is associated with tumor aggressiveness and worse disease-specific survival in colorectal cancer. Cancer Biomark. 2022;33(3):277–89. 34958000 10.3233/CBM-210330 PMC12364155

[20] Ding L, Yang Y, Ge Y, Lu Q, Yan Z, Chen X, et al. Inhibition of DCLK1 with DCLK1-IN-1 Suppresses Renal Cell Carcinoma Invasion and Stemness and Promotes Cytotoxic T-Cell-Mediated Anti-Tumor Immunity. Cancers (Basel). 2021;13:22. 10.3390/cancers13225729 PMC8616267 34830884

[21] Huang L, Li T, Huang X, Chen X, Ju Y, Xu L, et al. Tumor necrosis factor promotes doublecortin-like kinase 1 expression and cellular reprogramming in intestinal epithelial cells leading to neoplastic transformation. Cell Commun Signal. 2025;23(1):415. 41039610 10.1186/s12964-025-02400-y PMC12492607

[22] Dogra S, Elayapillai SP, Qu D, Pitts K, Filatenkov A, Houchen CW, et al. Targeting doublecortin-like kinase 1 reveals a novel strategy to circumvent chemoresistance and metastasis in ovarian cancer. Cancer Lett. 2023;578:216437. 37838282 10.1016/j.canlet.2023.216437 PMC10872611

[23] Mirsky PO, Wagner PL, Mandic-Popov M, Donnenberg VS, Donnenberg AD. The Tumor Environment in Peritoneal Carcinomatosis and Malignant Pleural Effusions: Implications for Therapy. Cancers (Basel). 2025;17:19. 10.3390/cancers17193217 PMC12523412 41097743

[24] Campeau E, Ruhl VE, Rodier F, Smith CL, Rahmberg BL, Fuss JO, et al. A versatile viral system for expression and depletion of proteins in mammalian cells. PLoS ONE. 2009;4(8):e6529. 19657394 10.1371/journal.pone.0006529 PMC2717805

[25] Murthy D, Attri KS, Shukla SK, Thakur R, Chaika NV, He C, et al. Cancer-associated fibroblast-derived acetate promotes pancreatic cancer development by altering polyamine metabolism via the ACSS2-SP1-SAT1 axis. Nat Cell Biol. 2024;26(4):613–27. 38429478 10.1038/s41556-024-01372-4 PMC11021164

[26] Francavilla C, Lupia M, Tsafou K, Villa A, Kowalczyk K, Rakownikow Jersie-Christensen R, et al. Phosphoproteomics of Primary Cells Reveals Druggable Kinase Signatures in Ovarian Cancer. Cell Rep. 2017;18(13):3242–56. 28355574 10.1016/j.celrep.2017.03.015 PMC5382236

[27] Velletri T, Villa CE, Cilli D, Barzaghi B, Lo Riso P, Lupia M, et al. Single cell-derived spheroids capture the self-renewing subpopulations of metastatic ovarian cancer. Cell Death Differ. 2022;29(3):614–26. 34845371 10.1038/s41418-021-00878-w PMC8901794

[28] Fang Y, Xiao X, Wang J, Dasari S, Pepin D, Nephew KP, et al. Cancer associated fibroblasts serve as an ovarian cancer stem cell niche through noncanonical Wnt5a signaling. NPJ Precis Oncol. 2024;8(1):7. 38191909 10.1038/s41698-023-00495-5 PMC10774407

[29] Hu Y, Smyth GK. ELDA: extreme limiting dilution analysis for comparing depleted and enriched populations in stem cell and other assays. J Immunol Methods. 2009;347(1–2):70–8. 19567251 10.1016/j.jim.2009.06.008

[30] Neelakantan D, Dogra S, Devapatla B, Jaiprasart P, Mukashyaka MC, Janknecht R, et al. Multifunctional APJ Pathway Promotes Ovarian Cancer Progression and Metastasis. Mol Cancer Res. 2019;17(6):1378–90. 30858172 10.1158/1541-7786.MCR-18-0989 PMC6548659

[31] von Mering C, Jensen LJ, Snel B, Hooper SD, Krupp M, Foglierini M, et al. STRING: known and predicted protein-protein associations, integrated and transferred across organisms. Nucleic Acids Res. 2005;33(Database issue):D433–7. 15608232 10.1093/nar/gki005 PMC539959

[32] Davidowitz RA, Iwanicki MP, Brugge JS. In vitro mesothelial clearance assay that models the early steps of ovarian cancer metastasis. J Vis Exp. 2012(60):3888. 10.3791/3888 PMC3466628 22371143

[33] Iwanicki MP, Davidowitz RA, Ng MR, Besser A, Muranen T, Merritt M, et al. Ovarian cancer spheroids use myosin-generated force to clear the mesothelium. Cancer Discov. 2011;1(2):144–57. 22303516 10.1158/2159-8274.CD-11-0010 PMC3269166

[34] Caminear MW, Harrington BS, Kamdar RD, Kruhlak MJ, Annunziata CM. Disulfiram Transcends ALDH Inhibitory Activity When Targeting Ovarian Cancer Tumor-Initiating Cells. Front Oncol. 2022;12:762820. 35372040 10.3389/fonc.2022.762820 PMC8967967

[35] Burkhard KM, Semwal A, Johnson BK, Chu KC, Kranick RJ, Rayan M et al. Tumoroid model recreates clinically relevant phenotypes of high grade serous ovarian cancer (HGSC) cells, carcinoma associated fibroblasts, and macrophages. Acta Biomater. 2026;213:251–68. 10.1016/j.actbio.2026.02.005 41655669

[36] Chefetz I, Grimley E, Yang K, Hong L, Vinogradova EV, Suciu R, et al. A Pan-ALDH1A Inhibitor Induces Necroptosis in Ovarian Cancer Stem-like Cells. Cell Rep. 2019;26(11):3061–75. e6. 30865894 10.1016/j.celrep.2019.02.032 PMC7061440

[37] Kim JH, Park SY, Jeon SE, Choi JH, Lee CJ, Jang TY, et al. DCLK1 promotes colorectal cancer stemness and aggressiveness via the XRCC5/COX2 axis. Theranostics. 2022;12(12):5258–71. 35910805 10.7150/thno.72037 PMC9330537

[38] Yao J, Huang X, Sun Q, Zhao W, Weygant N, Fan X, et al. Hypoxic stimulation of DCLK1 transcription and alternative-promoter switching fuels tumor malignancy in clear cell renal cell carcinoma. Cell Death Dis. 2025;16(1):594. 40775208 10.1038/s41419-025-07916-2 PMC12332081

[39] Liu H, Yan R, Xiao Z, Huang X, Yao J, Liu J, et al. Targeting DCLK1 attenuates tumor stemness and evokes antitumor immunity in triple-negative breast cancer by inhibiting IL-6/STAT3 signaling. Breast Cancer Res. 2023;25(1):43. 37069669 10.1186/s13058-023-01642-3 PMC10108533

[40] Sarkar S, O’Connell MR, Okugawa Y, Lee BS, Toiyama Y, Kusunoki M, et al. FOXD3 Regulates CSC Marker, DCLK1-S, and Invasive Potential: Prognostic Implications in Colon Cancer. Mol Cancer Res. 2017;15(12):1678–91. 28851816 10.1158/1541-7786.MCR-17-0287 PMC5748292

[41] O’Connell MR, Sarkar S, Luthra GK, Okugawa Y, Toiyama Y, Gajjar AH, et al. Epigenetic changes and alternate promoter usage by human colon cancers for expressing DCLK1-isoforms: Clinical Implications. Sci Rep. 2015;5:14983. 26447334 10.1038/srep14983 PMC4597220

[42] Guo T, Gu C, Li B, Xu C. PLODs are overexpressed in ovarian cancer and are associated with gap junctions via connexin 43. Lab Invest. 2021;101(5):564–9. 33483598 10.1038/s41374-021-00533-5

[43] Gong L, Zheng Y, Liu S, Peng Z. Fibronectin Regulates the Dynamic Formation of Ovarian Cancer Multicellular Aggregates and the Expression of Integrin Receptors. Asian Pac J Cancer Prev. 2018;19(9):2493–8. 30256042 10.22034/APJCP.2018.19.9.2493 PMC6249468

[44] Ahmed N, Riley C, Rice G, Quinn M. Role of integrin receptors for fibronectin, collagen and laminin in the regulation of ovarian carcinoma functions in response to a matrix microenvironment. Clin Exp Metastasis. 2005;22(5):391–402. 16283482 10.1007/s10585-005-1262-y

[45] Usui A, Ko SY, Barengo N, Naora H. P-cadherin promotes ovarian cancer dissemination through tumor cell aggregation and tumor-peritoneum interactions. Mol Cancer Res. 2014;12(4):504–13. 24448686 10.1158/1541-7786.MCR-13-0489 PMC3989397

[46] Kenny HA, Kaur S, Coussens LM, Lengyel E. The initial steps of ovarian cancer cell metastasis are mediated by MMP-2 cleavage of vitronectin and fibronectin. J Clin Invest. 2008;118(4):1367–79. 18340378 10.1172/JCI33775 PMC2267016

[47] Guo QY, Song JN, Chen YM, Yuan HN, Xue WS, Sun Y, et al. IL-6 regulates epithelial ovarian cancer EMT, invasion, and metastasis by modulating Let-7c and miR-200c through the STAT3/HIF-1alpha pathway. Med Oncol. 2024;41(6):155. 38744773 10.1007/s12032-024-02328-2

[48] Zhang T, Yang J, Sun Y, Song J, Gao D, Huang S, et al. Interleukin-6 and Hypoxia Synergistically Promote EMT-Mediated Invasion in Epithelial Ovarian Cancer via the IL-6/STAT3/HIF-1alpha Feedback Loop. Anal Cell Pathol (Amst). 2023;2023:8334881. 36814597 10.1155/2023/8334881 PMC9940980

[49] Niu N, Yao J, Bast RC, Sood AK, Liu J. IL-6 promotes drug resistance through formation of polyploid giant cancer cells and stromal fibroblast reprogramming. Oncogenesis. 2021;10(9):65. 34588424 10.1038/s41389-021-00349-4 PMC8481288

[50] Bourguignon LY, Peyrollier K, Xia W, Gilad E. Hyaluronan-CD44 interaction activates stem cell marker Nanog, Stat-3-mediated MDR1 gene expression, and ankyrin-regulated multidrug efflux in breast and ovarian tumor cells. J Biol Chem. 2008;283(25):17635–51. 18441325 10.1074/jbc.M800109200 PMC2427357

[51] Abubaker K, Luwor RB, Zhu H, McNally O, Quinn MA, Burns CJ, et al. Inhibition of the JAK2/STAT3 pathway in ovarian cancer results in the loss of cancer stem cell-like characteristics and a reduced tumor burden. BMC Cancer. 2014;14:317. 24886434 10.1186/1471-2407-14-317 PMC4025194

[52] Dadgar N, Sherry C, Zimmerman J, Park H, Lewis C, Donnenberg A, et al. Targeting interleukin-6 as a treatment approach for peritoneal carcinomatosis. J Transl Med. 2024;22(1):402. 38689325 10.1186/s12967-024-05205-8 PMC11061933

