# Supplementary material for: DCLK1 regulates stemness and IL-6/STAT3–dependent metastatic niche formation in chemoresistant ovarian cancer
Source: J Exp Clin Cancer Res. 2026 May 28;45:166. doi: 10.1186/s13046-026-03739-x (PMC13404908; doi:10.1186/s13046-026-03739-x)
Supplement: Supplementary file 1 — Supplementary Material 1. Supplementary Table S1. Clinicopathologic Characteristics of Ovarian Cancer Patients in this Study.Supplementary Table S2. Antibody dilutions used for Western blots.Supplementary Table S3. Primer sequences for qRT-PCRSupplementary Table S4. Differentially expressed cytokines/ chemokines.Supplementary Table S5. DCLK1 Expression in ovarian cancer patient-derived ascites cultures [file 13046_2026_3739_MOESM1_ESM.pdf]

Supplementary Table S1: Clinicopathologic Characteristics of Ovarian Cancer Patients in this Study

ID	Age	Diagnosis/ Histology	Disease Stage	Treatment Status (Prior to ascites collection)
Initial Samples				
ASC3	44	HGSOC	IIIC	Untreated
ASC6	49	HGSOC	IIIC	Untreated
ASC9	62	HGSOC	IIIC	Carboplatin/ Paclitaxel
ASC27	77	HGSOC	IVB	Carboplatin/ Paclitaxel
ASC28	61	HGSOC	IIIB	Carboplatin/ Paclitaxel/ Bevacizumab
ASC30	72	HGSOC	IVB	Untreated
ASC38	50	HGSOC	IVA	Carboplatin/ Doxorubicin/ Ipatasertib
ASC39	64	HGSOC	IIIC	Carboplatin/ Paclitaxel/ Bevacizumab
ASC42	74	HGSOC	IIIC	Untreated
ASC44	60	HGSOC	IIIC	Untreated
ASC48	78	HGSOC	IIIC	Untreated
Recurrent Samples				
ASC2	68	HGSOC	IIIC	Carboplatin/ Doxorubicin/ Bevacizumab
ASC4A	79	HGSOC	IVA	Carboplatin
ASC5	74	HGSOC	IIIC	IMGN151 (GYN 116)
ASC9B	62	HGSOC	IIIC	Carboplatin/ Paclitaxel
ASC21	76	HGSOC	IIIC	CDH6 antibody drug conjugate
ASC24	49	HGSOC	IVB	IMGN151 (GYN 116)
ASC25	76	HGSOC	IVB	Carboplatin
ASC32	69	HGSOC	IIIC	Carboplatin/ Paclitaxel/ Bevacizumab
ASC33	72	HGSOC	IIIC	Carboplatin/ Doxorubicin/ Bevacizumab
ASC41	51	HGSOC	IIIC	Mirvetuximab soravtansine/ Bevacizumab

Supplementary Table S2: Antibody dilutions used for Western blots

Antibody	Company	Catalog Number	Dilution Used
Conventional Western blot			
ALDH1A3	Invitrogen	PA5-29188	1:1000
CD133	Cell Signaling	64326S	1:1000
ALDH1A1	Cell Signaling	12035	1:1000
CD44	Cell Signaling	5640	1:1000
Vimentin	Cell Signaling	5741	1:1000
N-Cadherin	Cell Signaling	13116	1:1000
Slug	Cell Signaling	9585	1:1000
Snail	Cell Signaling	3879	1:1000
E-Cadherin	Cell Signaling	3195	1:1000
IL-6R	Cell Signaling	18935	1:1000
pJAK2	Cell Signaling	4406	1:1000
Total JAK2	Cell Signaling	3230	1:1000
pSTAT3	Cell Signaling	9145	1:1000
Total STAT3	Cell Signaling	12640	1:1000
GAPDH	Cell Signaling	97166S	1:1000
α-Tubulin	Cell Signaling	3873S	1:1000
β-Actin	Cell Signaling	4967S	1:1000
HRP-anti-rabbit IgG	Cell Signaling	7074S	1:2500
HRP-anti-mouse IgG	Cell Signaling	7076S	1:2500
ProteinSimple JESS			
EPCAM	Cell Signaling	2626	1:50
PAX8	Novus Biologicals	NBP1-32440	1:50
DCLK1	Abcam	ab31704	1:25
DCLK1	Abcam	ab109029	1:50
ALDH1A3	Invitrogen	PA5-29188	1:50
CD133	Cell Signaling	64326	1:25

Supplementary Table S3: Primer sequences for qRT-PCR

Gene	Forward	Reverse
KLF4 Human	GCAGCCACCTGGCGAGTCTG	CCGCCAGCGGTTATTCGGGG
Nanog Human	TGGGATTTACAGGCGTGAGCCAC	AAGCAAAGCCTCCCAATCCCAAAC
Oct-4 Human	GCAGCCACCTGGCGAGTCTG	CACCTTCCCTCCAACCAGTTGC
36B4 Human	ATCAACGGGTACAAACGAGTCCTG	AAGGCAGATGGATCAGCCAAGAAG

Supplementary Table S4: Differentially expressed cytokines/ chemokines

Gene	Fold-change (Relative to sgCtrl)	Gene	Fold-change (Relative to sgCtrl)
TGFB2	125.82	SPP1	1.05
HGF	11.02	CXCL10	1.05
IGFBP1	6.09	IL16	1.04
CXCL7	5.92	TNFRSF11B	1.01
CCL13	3.94	OSM	0.98
CCL24	3.16	IFNG	0.98
TGFB3	2.46	FGF6	0.97
PGF	2.31	TNFA	0.96
MIF	1.99	CCL2	0.96
GDNF	1.91	TIMP2	0.95
NTF3	1.88	CXCL12	0.93
IGF1	1.83	IL1B	0.92
CCL11	1.67	IL3	0.92
CCL23	1.63	IL7	0.91
CXCL13	1.57	KGF	0.91
LEP	1.56	CCL15	0.90
CXCL6	1.49	CCL22	0.88
TNFSF14	1.48	THPO	0.87
CCL18	1.43	VEGF	0.86
BDNF	1.43	IL12A	0.86
PDGFB	1.39	TIMP1	0.86
EGF	1.32	IL2	0.86
IGFBP4	1.30	IL4	0.83
CX3CL1	1.28	CSF1	0.82
FLT3LG	1.26	IL15	0.82
TGFB1	1.25	FGF9	0.81
FGF4	1.24	IL13	0.81
NTF4	1.21	KITLG	0.81
CCL17	1.20	CXCL1	0.80
IGFBP2	1.17	CCL5	0.78
IL10	1.16	CCL8	0.75
CCL1	1.15	IL5	0.65
CXCL8	1.14	CSF2	0.59
CCL26	1.10	CXCL5	0.53
LIF	1.10	CCL7	0.51
TNFSF1B	1.09	CXCL1	0.44
IGFBP3	1.08	CCL20	0.17
IL1A	1.07	IL6	0.10
ANG	1.07	CSF3	0.08
CXCL9	1.06		
CCL4	1.05		

Supplementary Table S5: Bioluminescence-based quantification of tumor burden

Week post-implantation	Total Emission (Photons/s) [Mean, SD]	
	OVCAR-8 CPR sgCtrl luc	OVCAR-8 CPR sgDCLK1#1 luc
Week 1	328805790.00	3574425.00
	194986960.00	3836408.00
	111596060.00	10624827.00
	94363432.00	1846426.30
	115592230.00	162948.67
Week 2	182552700.00	13279018.00
	69406888.00	2656499.30
	30009354.00	8266014.00
	6676181.00	3333685.80
	47882056.00	2004226.50
Week 3	573728830.00	59002728.00
	103021330.00	40740576.00
	57061728.00	44126564.00
	70123632.00	23238598.00
	228998000.00	11569274.00
Week 4	1325996300.00	121027510.00
	542976380.00	6821062.00
	204075660.00	70869648.00
	200671220.00	81026048.00
	924296260.00	81208216.00
Week 5	2184707800.00	223381460.00
	1948302300.00	148339180.00
	76305136.00	107538750.00
	1466406700.00	164098880.00
	1637606500.00	250870910.00

Supplementary Table S6: DCLK1 Expression in ovarian cancer patient-derived ascites cultures

ID (Initial samples)	Total DCLK1 Expression (Normalized Peak Area)	ID (Recurrent samples)	Total DCLK1 Expression (Normalized Peak Area)
ASC3	112415.2	ASC2	0
ASC6	246596.2	ASC4A	1520193
ASC9	608.3	ASC5	0
ASC27	288193.9	ASC9B	1490952
ASC28	220630.7	ASC21	2068585
ASC30	0	ASC24	0
ASC38	2329260	ASC25	1406708
ASC39	38118.1	ASC32	4492199
ASC42	0	ASC33	592408.7
ASC44	712	ASC41	1745643
ASC48	371825.1		
