# Supplementary material for: DCLK1 regulates stemness and IL-6/STAT3–dependent metastatic niche formation in chemoresistant ovarian cancer
Source: J Exp Clin Cancer Res. 2026 May 28;45:166. doi: 10.1186/s13046-026-03739-x (PMC13404908; doi:10.1186/s13046-026-03739-x)
Supplement: Supplementary file 2 — Supplementary Material 2. Supplementary Figure 1. In vivo DCLK1 knockout has no significant effect on body weight in OC mouse modelsSupplementary Figure 2. Loss of DCLK1 reduces ALDH1A1/CD44 expression and ALDH activity.Supplementary Figure 3. DCLK1 expression is correlated with IL-6 secretion in patient-derived acellular ascites samples.Supplementary Figure 4. Paracrine IL-6 signaling activates mesothelial JAK–STAT3 signaling. [file 13046_2026_3739_MOESM2_ESM.pdf]

Supplementary Figures

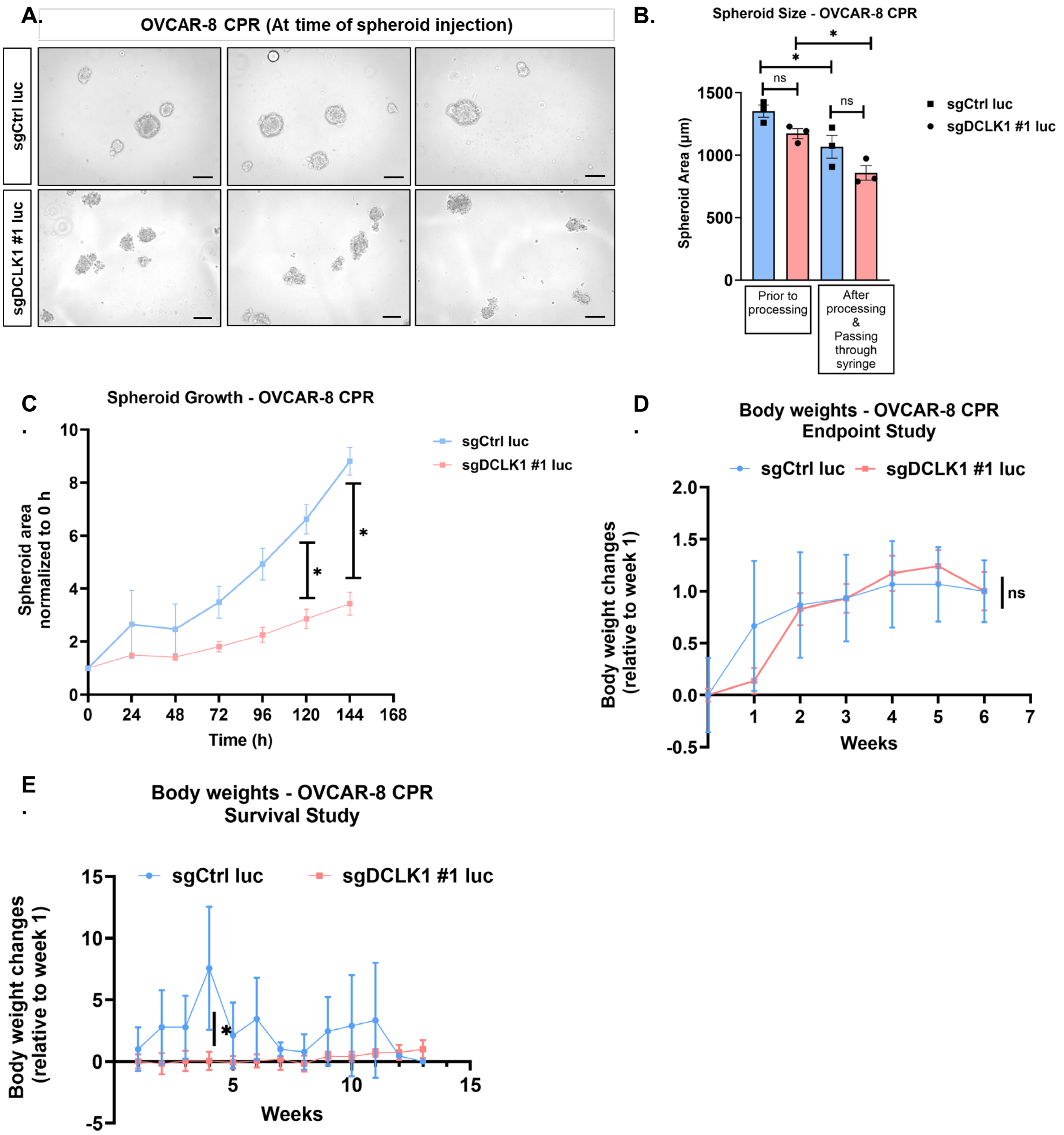

Supplementary Figure 1. In vivo DCLK1 knockout has no significant effect on body weight in OC mouse models. A. Representative images of OVCAR-8 CPR sgCtrl luc and sgDCLK1 #1 luc spheroids. B. Spheroid size quantifications of the luciferase-tagged DCLK1 knockout OVCAR-8 CPR spheroids prior to processing and after passing through syringe used in mouse experiments. C. Spheroid growth of OVCAR-8 CPR sgCtrl luc and sgDCLK1 #1 luc cells measured using IncuCyte live-cell imaging system. D, E. Normalized weights of female athymic nude mice that received intraperitoneal injections of OVCAR-8 CPR sgCtrl luc and sgDCLK1 #1 luc spheroids, used in the (D) endpoint study, and (E) survival study. Statistical analysis was performed using one-way ANOVA followed by Tukey's multiple comparison test in B, RM two-way ANOVA followed by Tukey's multiple comparison test in C-E. *P<0.05; ns: not significant. CPR: cisplatin resistant.

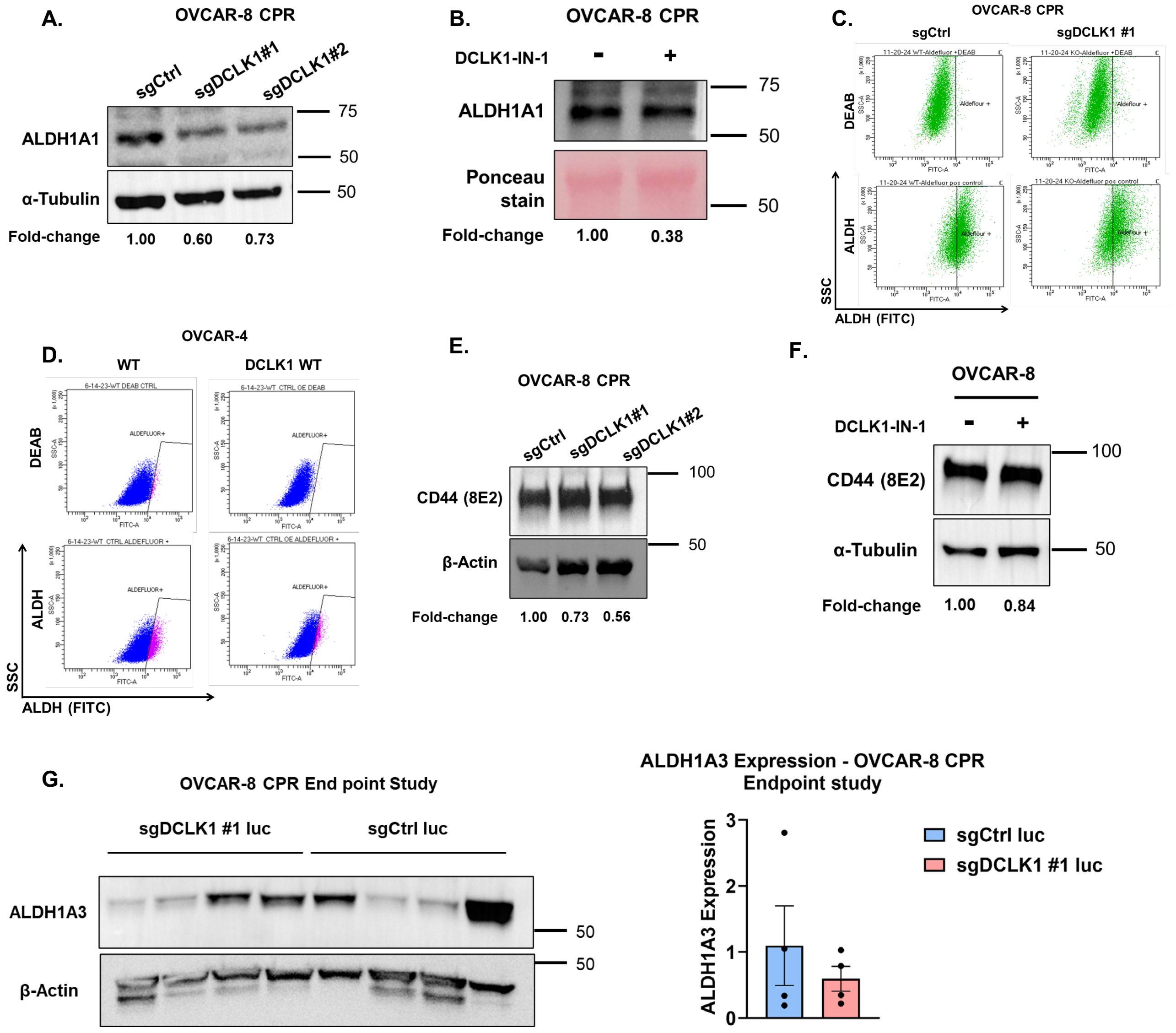

Supplementary Figure 2. Loss of DCLK1 reduces ALDH1A1/CD44 expression and ALDH activity. A, B. Immunoblot of ALDH1A1 expression in (A) OVCAR-8 CPR DCLK1 knockout spheroids and (B) OVCAR-8 CPR spheroids treated with DCLK1-IN-1. C, D. Representative flow cytometry density plots from the Aldefluor™ assay showing ALDH+ populations for (C) OVCAR-8 CPR DCLK1 knockout cells and (D) OVCAR-4 DCLK1 over-expression cells. DEAB-treated samples serve as negative controls to establish gating. E, F. Representative western blots of CD44 expression in (E) OVCAR-8 CPR DCLK1 knockout spheroids and (F) OVCAR-8 CPR spheroids treated with DCLK1-IN-1. G Representative western blot of ALDH1A3 expression in tumor tissue lysates derived from the OVCAR-8 CPR DCLK1 knockout end point study and its quantification. These results were obtained from ≥3 independent experiments in A-F.

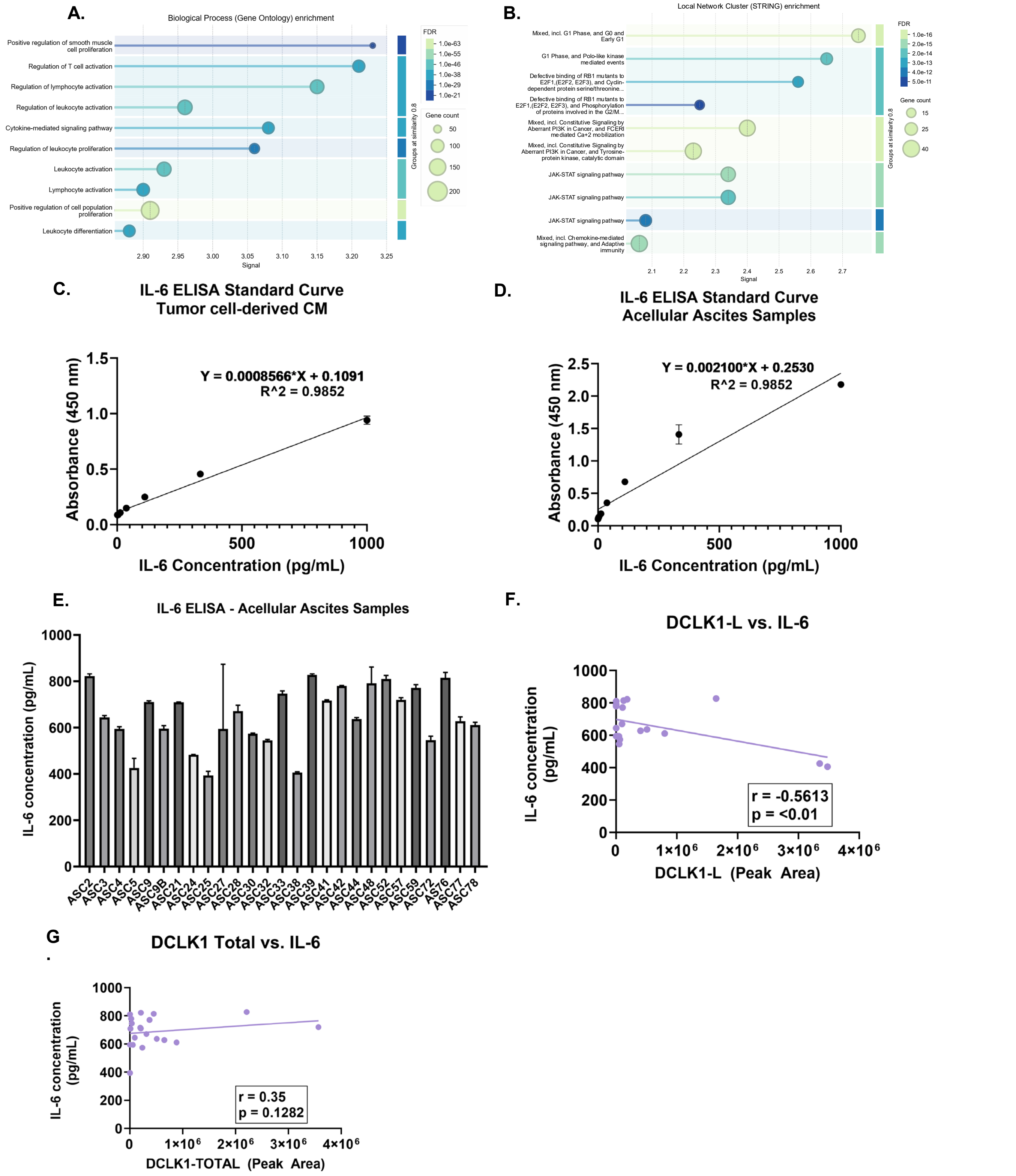

Supplementary Figure 3. DCLK1 expression is correlated with IL-6 secretion in patient-derived acellular ascites samples. . A, B. Differentially expressed genes from control and DCLK1-knockout spheroids analyzed using (A) Gene Ontology and (B) STRING enrichment tools. C,D. Standard curves generated for human IL-6 ELISA using serially diluted recombinant IL-6 standards (0–1,000 pg/mL). Absorbance values (450 nm) were plotted against known concentrations and fitted using a 4-parameter logistic curve to ensure accurate quantification for IL-6 in the (C) tumor cell-derived conditioned media (n=5) and (D) acellular ascites samples (n=28). E. Interpolated IL-6 concentrations in acellular ascites samples derived from initial (n=17) and recurrent (n=11) OC patients. F, G Scatter plot depicting the correlation between (F) DCLK1-L, and (G) DCLK1 total expression and IL-6 secretion. Pearson's correlation coefficient (r) was computed in F, G. Correlation strength was interpreted as: weak (0.1–0.3), moderate (0.3–0.5), and strong (>0.5).

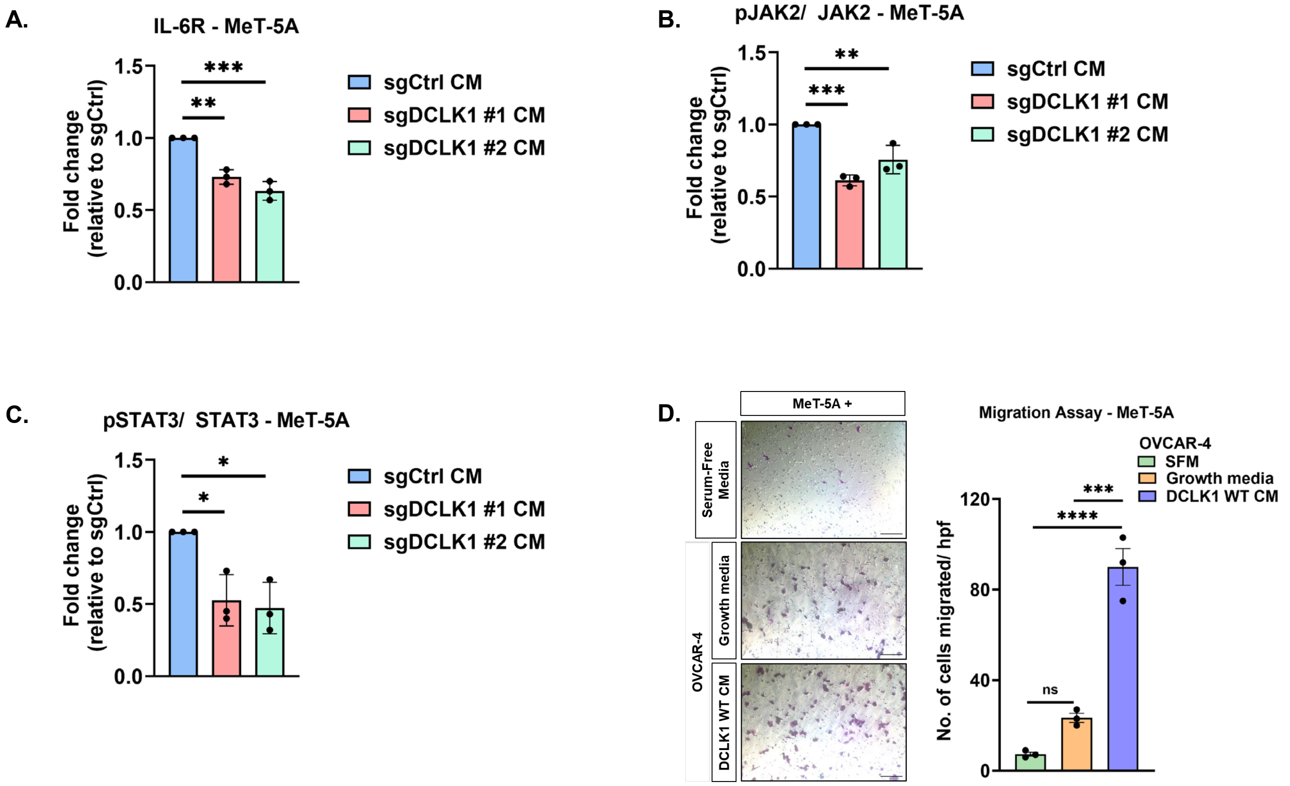

Supplementary Figure 4. Paracrine IL-6 signaling activates mesothelial JAK–STAT3 signaling. A-C Quantification of western blot analysis of IL-6/STAT3 pathway proteins in MeT-5A cell lysates treated with conditioned media collected from sgCtrl or DCLK1-knockout ovarian cancer spheroids. Quantified proteins include (A) IL-6 receptor, (B) pJAK2/JAK2, and (C) pSTAT3/STAT3. D. Representative images of MeT-5A cell migration in the presence of serum-free media, growth media containing 10% FBS, or conditioned media derived from OVCAR-4 spheroids overexpressing wild-type DCLK1. These results were obtained from ≥3 independent experiments in A-D. Statistical analysis was performed using one-way ANOVA followed by Tukey's multiple comparison test in A-D. *P<0.05; **p<0.01; ***p<0.001; ****p<0.0001; ns: not significant. CM, conditioned-media; DCLK1 WT: Over-expression of wild-type DCLK1.
